# Mutation update on *ACAT1* variants associated with mitochondrial acetoacetyl‐CoA thiolase (T2) deficiency

**DOI:** 10.1002/humu.23831

**Published:** 2019-07-03

**Authors:** Elsayed Abdelkreem, Rajesh K. Harijan, Seiji Yamaguchi, Rikkert K. Wierenga, Toshiyuki Fukao

**Affiliations:** ^1^ Department of Pediatrics, Graduate School of Medicine Gifu University Gifu Japan; ^2^ Department of Pediatrics, Faculty of Medicine Sohag University Sohag Egypt; ^3^ Department of Biochemistry Albert Einstein College of Medicine New York New York; ^4^ Department of Pediatrics Shimane University School of Medicine Izumo Japan; ^5^ Biocenter Oulu and FBMM University of Oulu Oulu Finland

**Keywords:** *ACAT1*, genotype‐phenotype correlation, mutations, structure, T2‐deficiency, variants, β‐ketothiolase deficiency

## Abstract

Mitochondrial acetoacetyl‐CoA thiolase (T2, encoded by the *ACAT1* gene) deficiency is an inherited disorder of ketone body and isoleucine metabolism. It typically manifests with episodic ketoacidosis. The presence of isoleucine‐derived metabolites is the key marker for biochemical diagnosis. To date, 105 *ACAT1* variants have been reported in 149 T2‐deficient patients. The 56 disease‐associated missense *ACAT1* variants have been mapped onto the crystal structure of T2. Almost all these missense variants concern residues that are completely or partially buried in the T2 structure. Such variants are expected to cause T2 deficiency by having lower in vivo T2 activity because of lower folding efficiency and/or stability. Expression and activity data of 30 disease‐associated missense *ACAT1* variants have been measured by expressing them in human SV40‐transformed fibroblasts. Only two variants (p.Cys126Ser and p.Tyr219His) appear to have equal stability as wild‐type. For these variants, which are inactive, the side chains point into the active site. In patients with T2 deficiency, the genotype does not correlate with the clinical phenotype but exerts a considerable effect on the biochemical phenotype. This could be related to variable remaining residual T2 activity in vivo and has important clinical implications concerning disease management and newborn screening.

## 1 INTRODUCTION

The mitochondrial acetoacetyl‐CoA thiolase (commonly known as β‐ketothiolase [T2]; EC 2.3.1.9; encoded by the *ACAT1* gene) is a ubiquitous and important enzyme for ketone body synthesis and degradation as well as in isoleucine catabolism (Fukao et al., 2014, 2018). Human tissues have, at least, five other thiolase isoenzymes: Cytosolic acetoacetyl‐CoA thiolase (CT, EC 2.3.1.9), mitochondrial 3‐ketoacyl‐CoA thiolase (T1, EC 2.3.1.16), the β subunit of the mitochondrial trifunctional enzyme that catalyzes 3‐ketoacyl‐CoA thiolase activity (TFE, EC 2.3.1.16), the peroxisomal 3‐ketoacyl‐CoA thiolase (AB‐thiolase, EC 2.3.1.16), and the peroxisomal thiolase type‐1 (SCP2‐thiolase; EC 2.3.1.176). These thiolases (excluding the SCP2‐thiolase) share 35–46% sequence identity and have both synthetic and degradative functions; the degradative SCP2‐thiolase has very low sequence similarity with any of the other thiolase family members. These thiolases are either dimers (tight dimers) or tetramers (dimers of tight dimers) (Fukao, 2002; Harijan et al., 2013; Kiema et al., 2019).

**Figure 1:**
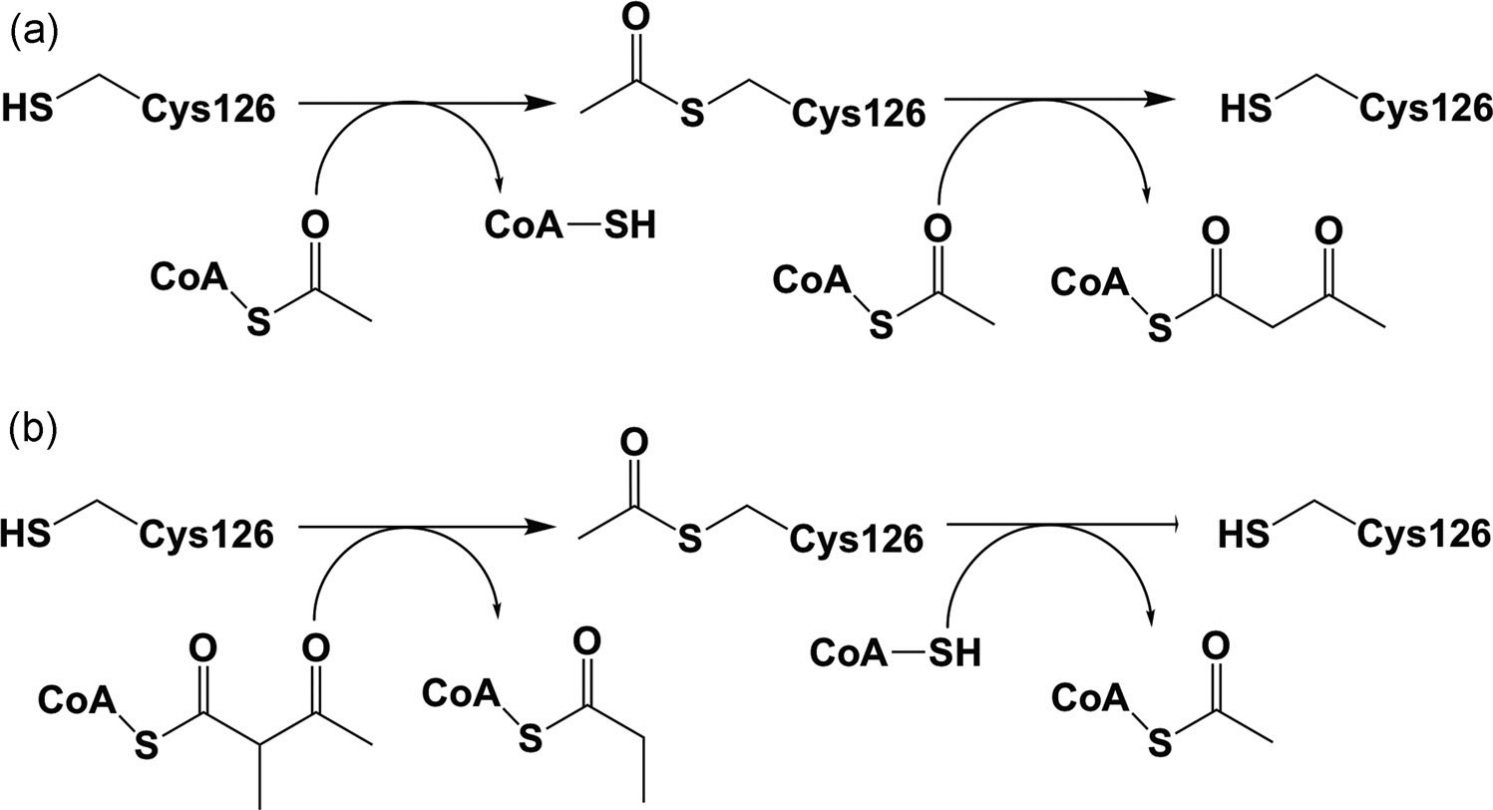
The reactions catalyzed by the T2 thiolase. (a) The biosynthetic reaction: The substrates are two molecules of acetyl‐CoA. (b) The degradative reaction: The substrates are 2‐methylacetoacetyl‐CoA (or acetoacetyl‐CoA) and CoA. In both directions, the reaction mechanism proceeds via a covalent intermediate, in which the nucleophilic cysteine, Cys126 in human T2, becomes acetylated in the biosynthetic as well as in the degradative reactions

**Figure 2:**
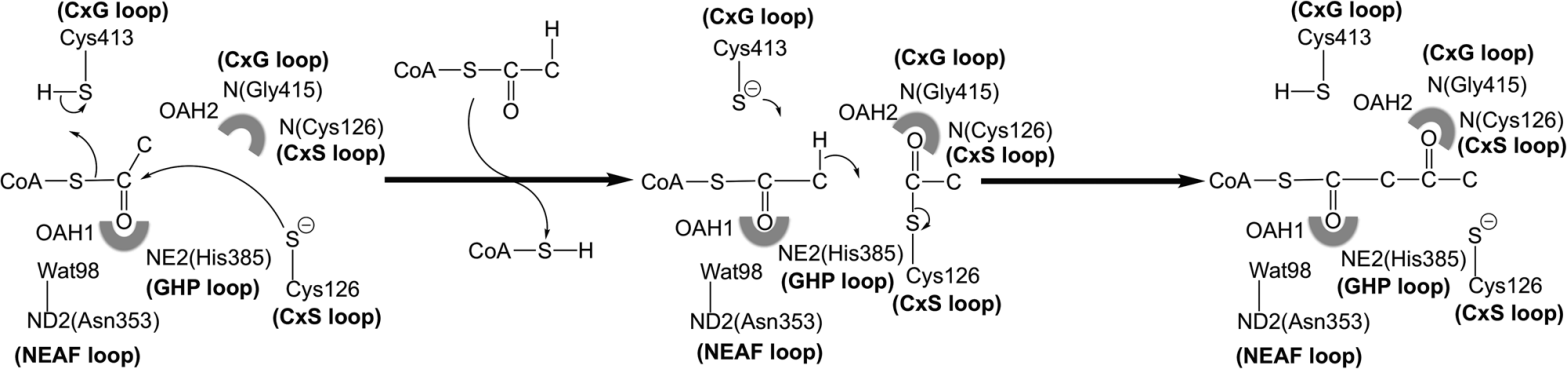
Schematic drawing showing the T2 thiolase reaction in the synthetic direction. Two molecules of acetyl‐CoA are converted into CoA and acetoacetyl‐CoA. The role of the four catalytic residues (Cys126, Asn353, His385, Cys413 of human T2) is highlighted. These residues protrude into the catalytic site from the four catalytic loops (the CxS, NEAF, GHP, and CxG loops, respectively, shown in bold). Cys126 is the nucleophilic cysteine and Cys413 is the acid/base cysteine. The side chains of Asn353 (fixing Wat98) and His385, as well as the main chain *N*‐atoms of the CxS and CxG loops, contribute to the two oxyanion holes (OAH1 and OAH2, shown as shaded semicircles). These oxyanion holes stabilize the negative charge that develops during the reaction on the thioester oxygen atom of the reaction intermediates, being therefore also critically important for catalysis. The short‐curved arrows visualize the breaking/forming of bonds

**Figure 3:**
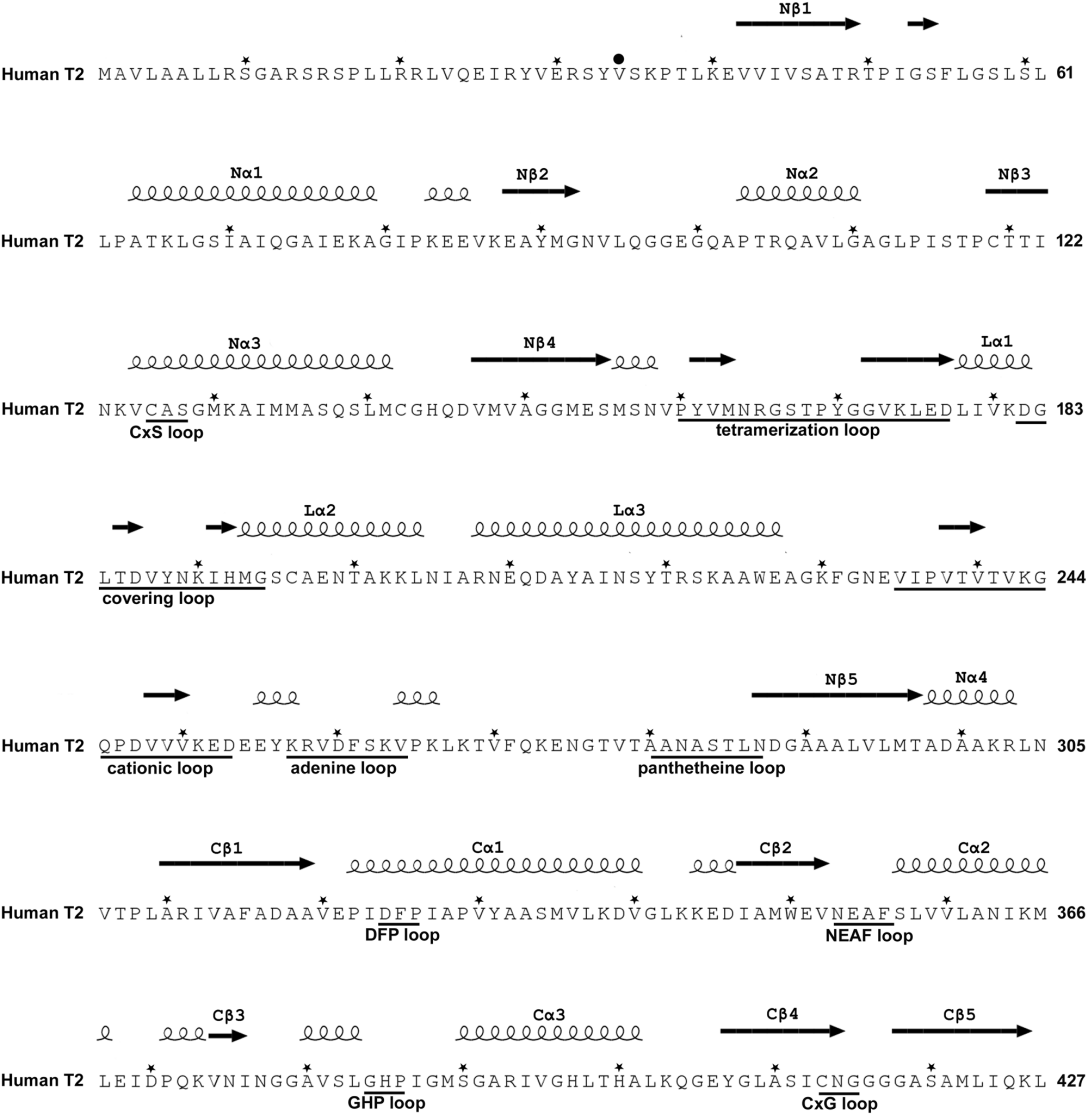
The sequence of the human mitochondrial acetoacetyl‐CoA thiolase (T2, UniProt code: P24752) with nomenclature of secondary structure, sequence fingerprints, and loops. The *N*‐terminal region is the mitochondrial leader sequence, which is cleaved off on entry into the mitochondria. The secondary structure is obtained from the structure of the human T2 (PDB code: 2IBW) using the ESPript 3.0 server (Robert & Gouet, 2014) and shown above the sequence. An asterisk (*) above the sequence marks every tenth residue. The mature sequence starts at Val34, indicated by a black circle (•) above the sequence. Important active site loops that are near the catalytic site are identified below the sequence with their sequence fingerprint. The nomenclature of the functional regions of the loop domain (residues 156–286) is also given below the sequence. The structural properties of the latter loop regions are visualized in Figure 7 and Figure S3

In the biosynthetic direction, thiolases catalyze the formation of a carbon‐carbon bond through a Claisen condensation mechanism (from two acetyl‐CoA molecules) and in the reverse, degradative direction a C‐C bond is broken through thiolysis (in the presence of CoA), resulting in chain shortening of the acyl chain by two carbon atoms (in case the substrate is an unbranched acyl chain) or by three atoms (in case the substrate is a 2‐methyl‐branched acyl chain), such as for example catalyzed by the T2 (Figure 1; Haapalainen, Meriläinen, & Wierenga, 2006; Song et al., 1994). No cofactors are required for the catalytic activity of thiolases, and each thiolase catalyzes the reaction in both directions. The crystal structures of several thiolases have been reported (Haapalainen et al., 2006; Kiema et al., 2019). From this structural information as well as from extensive sequence alignment, a classification of thiolases has been proposed (Anbazhagan et al., 2014). The crystal structure of the wild‐type human T2 thiolase tetramer has been reported in 2007 (Haapalainen et al., 2007). Two cysteines are important for catalysis. The nucleophilic cysteine (Cys126 in human T2 thiolase) becomes acetylated in the reaction cycle (Figure 1), whereas the second catalytic cysteine (Cys413) functions as an acid/base (Figure 2). These cysteines protrude into the catalytic site from two different catalytic loops, being the CxS loop and the CxG loop (Figure 2 and Figure 3).

Ketone bodies (acetoacetate and 3‐hydroxybutyrate) are important energy sources for most tissues, particularly the brain. Ketone body synthesis begins in the liver by β‐oxidation of free fatty acids to output acetyl‐CoA and acetoacetyl‐CoA. T2 in the liver catalyzes the Claisen condensation of two acetyl‐CoA molecules into acetoacetyl‐CoA. In extrahepatic tissues, T2 is responsible for thiolytic cleavage of acetoacetyl‐CoA into two molecules of acetyl‐CoA. T2 deficiency causes episodic ketoacidosis. This indicates that T2 deficiency impedes ketolysis to a greater extent than ketogenesis. The abundant amount of T1 in the liver likely compensates for T2 deficiency in ketogenesis (Fukao et al., 2014). Potassium ions specifically enhance the activity of T2 but do not change that of T1 and other thiolases, therefore the potassium ion‐activated acetoacetyl‐CoA thiolase assay remains the gold‐standard test for the T2 enzyme assay (Middleton, 1973).

In isoleucine catabolism, T2 catalyzes the thiolysis of 2‐methylacetoacetyl‐CoA (2MAA‐CoA) to acetyl‐CoA and propionyl‐CoA. T2 deficiency is characterized by excessive accumulation of isoleucine‐catabolic intermediates that can be detected in urine as 2‐methylacetoacetate (2MAA), 2‐methyl‐3‐hydroxybutyrate (2M3HB), and tiglyl‐glycine (TIG) and in blood as tiglyl‐carnitine and 2M3HB‐carnitine; notably, 2MAA is rapidly degraded and, consequently, is sometimes hardly detected in urine samples, especially in nonfresh ones (Aramaki et al., 1991). Therefore, T2 deficiency results in excessive accumulation of not only 2MAA‐CoA but also of the two upstream metabolites, namely 2M3HB‐CoA and 2‐methyl‐2E‐butenoyl‐CoA (tiglyl‐CoA) (Fukao et al., 2014).

T2 deficiency (MIM# 203750, 607809) is an autosomal recessive disease. Deficiencies of T2 and 3‐hydroxy‐3‐methylglutaryl‐CoA lyase (EC 4.1.3.4; MIM# 246450) constitute the most common inborn errors of ketone body metabolism (Abdelkreem et al., 2016; Fukao et al., 2014). Since Daum, Lamm, Mamer, and Scriver (1971), for the first time, characterized T2 deficiency, at least 159 patients (Supporting Information Table) with the disease have been confirmed (through enzyme assay and/or genetic analysis) worldwide without ethnic preference. The incidence of T2 deficiency has been estimated in some regions, as one per 232,000 newborns in Minnesota, one per 190,000 newborns in northern Vietnam, and one per 111,000 newborns in Hyderabad (India) (Abdelkreem, Akella, et al., 2017; Nguyen et al., 2017; Sarafoglou et al., 2011).

Herein, we review 105 *ACAT1* variants that have been reported in 149 patients with T2 deficiency; we use the term “disease‐associated *ACAT1* variants” to refer to variants associated with T2 deficiency. A discussion on non‐disease‐associated *ACAT1* variants is beyond the scope of this review. We discuss important structural features of human T2 and the location of the disease‐associated missense *ACAT1* variants in the context of the crystal structure of human T2. To increase the understanding of this rare disease, we also discuss its clinical and laboratory implications.

## 2 THE T2 GENE AND DISEASE‐ASSOCIATED VARIANTS

The human *ACAT1* gene (NCBI reference sequence: NG_009888.1) is located on chromosome 11q22.3‐q23.1, spanning approximately 27 kb. This gene contains 12 exons interspersed by 11 introns. The 5ʹ‐flanking region lacks a classic TATA box, but it contains two CAAT boxes and is GC rich. These features are characteristic of housekeeping genes. Human T2 complementary DNA (cDNA; NCBI reference sequence: NM_000019.3) spans about 1.5 kb. It encodes a precursor protein (NCBI reference sequence: NP_000010.1) composed of 427 amino acids, including a leader polypeptide of 33‐amino acid (Kano et al., 1991).

**Figure 4:**
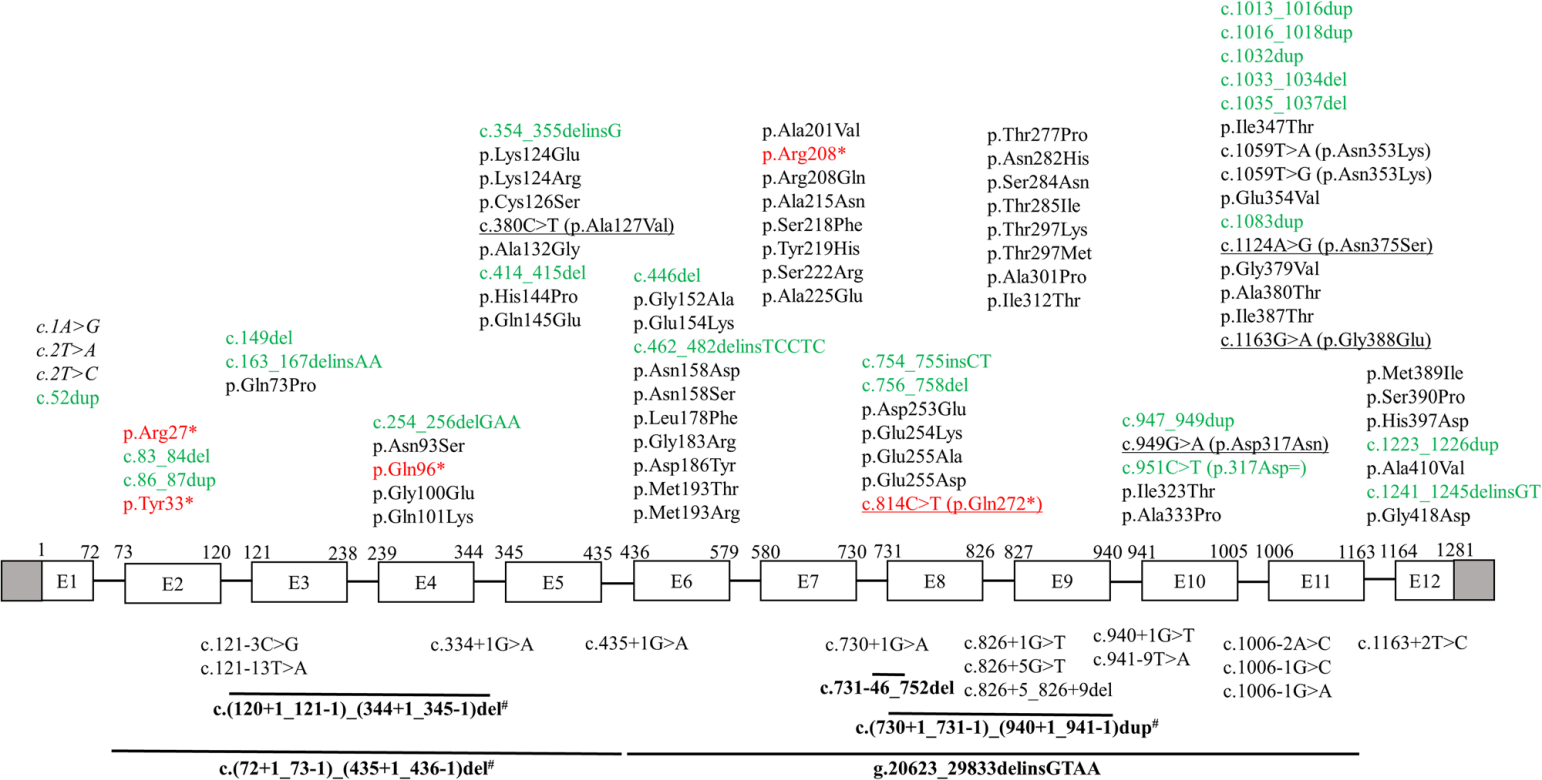
Schematic illustration (not to scale) of human *ACAT1* gene showing the location of 105 variants associated with mitochondrial acetoacetyl‐CoA thiolase deficiency. Exons (boxes) and introns (lines) are numbered according to NCBI refseq: NG_009888.1. Shaded boxes denote the untranslated region. Numbering of complementary DNA (cDNA; above boxes) is according to NCBI refseq: NM_000019.3, with +1 as the number of A of the ATG initiation codon. Description of variants follows the HGVS nomenclature (version 15.11, http://varnomen.hgvs.org; den Dunnen et al., 2016). Missense and nonsense variants are mainly described at the protein level (NCBI refseq: NP_000010.1). Exonic variants are shown above the diagram in black (missense), red (nonsense), and green (others); those associated with aberrant splicing are underlined, and those affecting the ATG initiation codon, causing reduced translation efficiency, are shown in italics. Intronic and large deletions/insertions/duplications variants are shown below the diagram. Large deletions/insertions/duplications are shown in bold with a solid line (‒) above depicting the approximate location. A number sign (^#^) marks variants attributed to *Alu*‐mediated unequal homologous recombination

The sequence of human T2 is shown in Figure 3. The available data on *ACAT1* variants associated with T2 deficiency are shown in three tables. Table 1, 2 have the information on the disease‐associated missense variants. These two tables also describe information on the location of the variant site with respect to the structure, in particular, whether the side chain of a residue is buried or whether it is exposed to bulk solvent. Table 1 lists the missense variants that have also been characterized with respect to (a) expression efficiency and (b) catalytic activity properties. The experimental details related to these characterizations are provided in the Supporting Information. For some variants, this information is available for expression at three temperatures; 30, 37, and 40°C. As can be seen in Table 1, there is generally a good correlation between the results obtained at different temperatures (e.g., whenever the data of expression at three temperatures are available, then the expression levels are the highest at 30°C and the lowest at 40°C). In addition, the activity recovery is generally never higher than the expression recovery. Table 3 lists other disease‐associated variants (ATG initiation codon, insertions, deletions, duplications, nonsense and aberrant splicing). Figure 4 depicts the location of the disease‐associated variants with respect to the exons of the *ACAT1* gene.

**Table 1:** Missense *ACAT1* variants associated with T2 deficiency, with available expression and activity data (*n* = 30)

E/I	Nucleotide change^a^	Predicted amino acid change^a^	In silico prediction of pathogenicity		Enzyme assay^d^/Expression assay^e^ by expression at			References	Comments on the structural information	Involvement of glycine or proline in the mutation	Important properties of each residue with respect to the structure of the tetramer^f^:
			PolyPhen‐2 score^b^	SIFT score^c^	37°C (in bold if equal or higher than 25% expressed)	30°C	40°C				
											‐Buried (completely buried)
											‐Surface (partially buried)
											‐Exposed side chain (side chain points towards solvent)
**E3**	c.218A>C	p.Gln73Pro	0.72	0.03	0%/0%	0%/0%	0%/0%	Sakurai et al. (2007)		Q73P	Surface
**E4**	c.278A>G	p.Asn93Ser	0.85	0.04	**8%/60%**	NM	NM	Fukao et al. (1998), Fukao, Zhang, et al. (2003)	At the dimer interface, maybe expressed as a folded monomer?		Buried
**E5**	c.371A>G	p.Lys124Arg	1.00	0.03	0%/0%	0%/0%	NM	Fukao et al. (2001), Fukao, Nakamura, et al. (2002)	At the dimer interface		Buried
**E5**	c.377G>C	p.Cys126Ser	0.99	0.00	**0%/100%**	NM	NM	This paper	Side chain points towards the catalytic site		Exposed side chain
**E5**	c.380C>T	p.Ala127Val (6% of mRNA), activates cryptic splice acceptor site causing c.336_386 del (p.Leu113_Gly129 del) (94% of mRNA)	0.98	0.01	0%/12%	0%/50%	NM	Nakamura et al. (2001), Fukao, Nakamura, et al. (2002)			Buried
**E5**	c.395C>G	p.Ala132Gly	**0.11**	0.02	10%/10%	25%/25%	NM	Zhang et al. (2004)		A132G	Buried
**E5**	c.431A>C	p.His144Pro	0.47	**0.23**	**25%/50%**	NM	25%/NM	Fukao et al. (2012)	At the dimer interface, maybe expressed as a folded monomer?	H144P	Surface
**E5**	c.433C>G	p.Gln145Glu	0.98	**0.76**	15%/12%	30%/25%	NM	Riudor et al. (1995), Fukao et al. (2001), Fukao, Nakamura, et al. (2002)	At the dimer interface, maybe expressed as a folded monomer?		Surface
**E6**	c.455G>C	p.Gly152Ala	1.00	0.00	0%/0%	5%/25%	NM	Zhang et al. (2004), Fukao et al. (2001), Fukao, Nakamura, et al. (2002), Buhaş et al (2013), Paquay et al. (2017)		G152A	Buried
**E6**	c.472A>G	p.Asn158Asp	0.93	0.05	0%/5%	0%/50%	0%/0%	Wakazono et al. (1995), Fukao, Yamaguchi, et al. (1995); Fukao et al. (2001), Sakurai et al. (2007), Buhaş et al. (2013), Otsuka et al. (2016)	At the dimer interface		Surface
**E6**	c.473A>G	p.Asn158Ser	0.80	**0.12**	0%/2%	0% / 3%	0%/0%	Sakurai et al. (2007), Sarafoglou et al. (2011)	At the dimer interface		Surface
**E6**	c.556G>T	p.Asp186Tyr	1.00	0.00	**0%/33%**	0%/NM	NM	Fukao, Horikawa, et al. (2010), Hori et al. (2015)	In the covering loop		Buried
**E6**	c.578T>G	p.Met193Arg	0.99	0.00	0%/0%	0%/0%	0%/0%	Ali et al. (2011), Akella et al. (2014), Abdelkreem, Akella, et al. (2017), Grünert et al. (2017)	Side chain points towards the pantetheine binding tunnel.		Exposed side chain
**E7**	c.623G>A	p.Arg208Gln	1.00	0.00	**0%/50%**	0%/60%	0%/50%	Sakurai et al. (2007)	Side chain fixes the adenine binding loop		Exposed side chain
**E7**	c.643_644delinsAA	p.Ala215Asn	1.00	0.00	0%/0%	0%/0%	0%/0%	Abdelkreem, Akella, et al. (2017)			Buried
**E7**	c.655T>C	p.Tyr219His	1.00	**0.13**	**0%/100%**	0%/100%	0%/50%	Fukao et al. (2001), Sakurai et al. (2007)	Side chain interacts with the potassium ion and the CoA moiety		Exposed side chain
**E7**	c.674C>A	p.Ala225Glu	1.00	0.00	0%/0%	NM	NM	Abdelkreem, Alobaidy, et al. (2017)			Surface
**E8**	c.759T>A	p.Asp253Glu	1.00	**0.35**	0%/0%	NM	NM	Fukao et al. (2001), this paper	Near the cationic loop		Buried
**E9**	c.844A>C	p.Asn282His	1.00	0.00	**0%/50%**	0%/100%	0%/40%	Sakurai et al. (2007)	In the pantetheine loop		Buried
**E9**	c.890C>T	p.Thr297Met	1.00	0.00	10%/12%	20%/20%	NM	Wakazono et al. (1995), Fukao et al. (2001), Zhang et al. (2004)			Surface
**E9**	c.901G>C	p.Ala301Pro	0.99	0.02	0%/10%	NM	NM	Wakazono et al. (1995)		A301P	Buried
**E9**	c.935T>C	p.Ile312Thr	0.96	0.00	8%/8%	NM	NM	Fukao et al. (1998), (2001), Fukao, Zhang, et al. (2003)			Buried
**E10**	c.949G>A	p.Asp317Asn (≈20% of mRNA), affects ESE sequence causing exon 10 skipping (≈80% of mRNA)	1.00	0.05	0%/0%	NM	NM	Otsuka et al. (2016), Köse et al. (2016), Grünert et al. (2017), this paper			Surface
**E10**	c.968T>C	p.Ile323Thr	0.87	**0.67**	**20%/25%**	40%/40%	0%/0%	Abdelkreem, Akella, et al. (2017)	In the Cβ1‐Cα1 loop that shapes the binding pocket of the 2‐methyl group of the 2‐methylacetoacetyl‐CoA substrate and side chain interacts with the covering loop		Exposed side chain
**E10**	c.997G>C	p.Ala333Pro	0.99	0.01	0%/0%	NM	NM	Fukao et al. (1998), (2001), Fukao, Zhang, et al. (2003)		A333P	Surface
**E11**	c.1059T>A	p.Asn353Lys	1.00	0.00	0%/0%	0%/0%	0%/0%	Sakurai et al. (2007)	In the NEAF loop		Buried
**E11**	c.1061A>T	p.Glu354Val	1.00	0.00	0%/0%	0%/0%	NM	Fukao et al. (2001), Fukao, Nakamura, et al. (2002)	In the NEAF loop		Buried
**E11**	c.1124A>G	p.Asn375Ser (11% of mRNA), activates a cryptic splice donor site causing c.1120_1163del (89% of mRNA)	1.00	0.00	0%/0%	0%/0%	NM	Fukao et al. (2008), Abdelkreem, Akella, et al. (2017)			Buried
**E12**	c.1168T>C	p.Ser390Pro	1.00	0.00	0%/0%	NM	0%/NM	Fukao et al. (2012)		S390P	Buried
**E12**	c.1189C>G	p.His397Asp	0.99	**0.12**	0%/0%	0%/0%	NM	Zhang et al. (2004), Catanzano et al. (2010), Paquay et al. (2017)			Buried

Abbreviations: E, exon; ESE, exonic splicing enhancer; I, intron; mRNA, messenger RNA; NM, not measured; T2, mitochondrial acetoacetyl‐CoA thiolase

^a^ Description of nucleotide changes, exons/introns, and predicted amino acid change follows the HGVS nomenclature (version 15.11, http://varnomen.hgvs.org; den Dunnen et al., 2016) using *ACAT1* NCBI reference sequences (NM_000019.3, NG_009888.1, and NP_000010.1) with +1 as the number of the A of the ATG initiation codon.

^b^ PolyPhen‐2 (polymorphism phenotyping v2; http://genetics.bwh.harvard.edu/pph2/) is a tool that predicts the effect of an amino acid substitution on protein structure and function. Score ranges from 0 to 1; higher scores predict an increased possibility for a damaging effect. A predicted benign value is shown in bold.

^c^ SIFT (sorting intolerant from tolerant; https://sift.bii.a-star.edu.sg/) is a sequence homology‐based tool that predicts the effect (damaging if the score is ≤0.05 and tolerated if the score is >0.05) of an amino acid substitution on protein function. Seven predicted tolerant values (score >0.05) are shown in bold.

^d^ Percentage of catalytic activity with respect to wild‐type T2 control, using potassium‐activated acetoacetyl‐CoA thiolase assay (Supporting Information Material).

^e^ Percentage of expressed soluble protein with respect to wild‐type T2 control (Material S2).

^f^ The classification is from visual inspection of the tetramer. The PDB code of the reference structure is 2IBW. This structure is the complex of human T2‐thiolase complexed with CoA, K^+^ and Cl^−^. For the classification, the unliganded structure (without CoA, K^+^, Cl^−^) has been considered.

**Table 2:** Missense *ACAT1* variants associated with T2 deficiency, with no available expression and activity data (*n* = 26)

E/I	Nucleotide change^a^	Predicted amino acid change^a^	References	Comments on the structural information	Involvement of glycine or proline in the mutation	Important properties of each residue with respect to the structure of the tetramer^b^:
						‐Buried (completely buried)
						‐Surface (partially buried)
						‐Exposed side chain (side chain points towards solvent)
E4	c.299G>A	p.Gly100Glu	Wojcik et al. (2018)	At the dimer interface	G100Q	Surface
**E4**	c.301C>A	p.Gln101Lys	Grünert et al. (2017)	At the dimer interface		Surface
**E5**	c.370A>G	p.Lys124Glu	Grünert et al. (2017)	At the dimer interface		Buried
**E6**	c.460G>A	p.Glu154Lys	Ali et al. (2011)			Buried
**E6**	c.534G>T	p.Leu178Phe	Paquay et al. (2017)	At the dimer interface		Buried
**E6**	c.547G>A	p.Gly183Arg	Fukao, Yamaguchi, Orii, Schutgens, et al. (1992), Fukao et al. (2001), Grünert et al. (2017), Hu et al. (2017)	At the dimer interface	G183R	Buried
**E6**	c.578T>C	p.Met193Thr	Mrázová et al. (2005), Thümmler et al. (2010)	Side chain points towards the pantetheine binding tunnel		Exposed side chain
**E7**	c.602C>T	p.Ala201Val	Fukao et al. (2013)			Buried
**E7**	c.653C>T	p.Ser218Phe	Wen et al. (2016)			Buried
**E7**	c.664A>C	p.Ser222Arg	Vakili and Hashemian (2018)			Buried
**E8**	c.760G>A	p.Glu254Lys	Paquay et al. (2017)	Just after the cationic loop, side chain fixes the Nβ1‐Nα1 loop		Exposed side chin
**E8**	c.764A>C	p.Glu255Ala	Sundaram, Nair, Namboodhiri, and Menon (2018)	This residue is just after the cationic loop		Surface
**E8**	c.765A>T	p.Glu255Asp	Paquay et al. (2017)	This residue is just after the cationic loop		Surface
**E9**	c.829A>C	p.Thr277Pro	Su et al. (2017)		T277P	Surface
**E9**	c.851G>A	p.Ser284Asn	Nguyen et al. (2017)	In the pantetheine binding loop		Surface
**E9**	c.854C>T	p.Thr285Ile	Al‐Shamsi, Hertecant, Al‐Hamad, Souid, and Al‐Jasmi (2014)	In the pantetheine binding loop		Surface
E9	c.890C>A	p.Thr297Lys	Su et al. (2017)			Surface
**E11**	c.1040T>C	p.Ile347Thr	Mrázová et al. (2005), Grünert et al. (2017)			Buried
**E11**	c.1059T>G	p.Asn353Lys	Paquay et al. (2017)	Part of the NEAF motif		Buried
**E11**	c.1136G>T	p.Gly379Val	Fukao et al. (1994), (2001)		G379V	Buried
**E11**	c.1138G>A	p.Ala380Thr	Fukao et al. (1991), (2001)			Buried
E11	c.1160T>C	p.Ile387Thr	Wojcik et al. (2018)	Side chain points towards the catalytic site.		Exposed side chin
**E11**	c.1163G>A	p.Gly388Glu, splice donor site with probable exon 11 skipping	Paquay et al. (2017)		G388E	Buried
**E12**	c.1167G>A	p.Met389Ile	Paquay et al. (2017)			Buried
**E12**	c.1229C>T	p.Ala410Val	Nguyen et al. (2015), (2017)			Buried
**E12**	c.1253G>A	p.Gly418Asp	Grünert et al. (2017)	At the dimer interface, interacts with the chloride ion	G418D	Buried

Abbreviations: E, exon; I, intron; T2, mitochondrial acetoacetyl‐CoA thiolase

^a^ Description of nucleotide changes, exons/introns, and predicted amino acid change follows the HGVS nomenclature (version 15.11, http://varnomen.hgvs.org; den Dunnen et al., 2016) using *ACAT1* NCBI reference sequences (NM_000019.3, NG_009888.1, and NP_000010.1) with +1 as the number of the A of the ATG initiation codon.

^b^ The classification is from visual inspection of the tetramer. The PDB code of the reference structure is 2IBW. This structure is the complex of human T2‐thiolase complexed with CoA, K^+^ and Cl^−^. For the classification, the unliganded structure (without CoA, K^+^, Cl^−^) has been considered.

**Table 3:** Other *ACAT1* variants associated with T2 deficiency (*n* = 49)

E/I	Nucleotide change^a^	Predicted amino acid change^a^	Reference
**(A) ATG initiation codon (*n* = 3)**			
**E1**	c.1A>G	Reduced translation efficiency (11%)	Fukao, Matsuo, et al. (2003), Nguyen et al. (2017)
**E1**	c.2T>A	Reduced translation efficiency (7.4%)	Fukao et al. (1993), Fukao, Matsuo, et al. (2003)
**E1**	c.2T>C	Reduced translation efficiency (19%)	Fukao, Zhang, et al. (2003), Fukao, Matsuo, et al. (2003)
**(B) In‐frame deletions/insertions/duplications (*n* = 7)**			
**E3**	c.163_167delinsAA	p.Phe55_Leu56delinsLys	Fukao, Nguyen, et al. (2010), Nguyen et al. (2015), (2017)
**E4**	c.254_256del	p.Glu85del	Fukao, Nakamura, et al. (2002)
**E8**	c.756_758del	p.Glu252del	Sakurai et al. (2007)
**E10**	c.947_949dup	p.Ala316dup	Paquay et al. (2017)
**E11**	c.1016_1018dup	p.Asp339dup	Zhang et al. (2004), Paquay et al. (2017)
**E11**	c.1035_1037del	p.Glu345del	Sewell et al. (1998), Fukao et al. (2001)
**E12**	c.1241_1245delinsGT	p.Asn414_Gly415delinsSer	Gibson, Elpeleg, and Bennett (1996), this paper
**(C) Out‐of‐frame deletions/insertions/duplications, nonsense, aberrant splicing, others (*n* = 39)**			
**E1**	c.52dup	p.Leu18Profs*49	Zhang et al. (2004), Sarafoglou et al. (2011), Paquay et al. (2017)
**E2**	c.79A>T	p.Arg27*	Paquay et al. (2017)
**E2**	c.83_84del	p.Tyr28Cysfs*38	Fukao et al. (1997), Paquay et al. (2017), Su et al. (2017)
**E2**	c.86_87dup	p.Glu30Trpfs*11	Al‐Shamsi et al. (2014), Al‐Jasmi, Al‐Shamsi, Hertecant, Al‐Hamad, and Souid (2016)
**E2**	c.99T>A	p.Tyr33*	Fukao, Yamaguchi, et al. (1995), Fukao et al. (2001)
**I2**	c.121–3C>G	Splice acceptor site (probably exon 3 skipping)	Su et al. (2017)
**I2**	c.121–13T>A	Splice acceptor site (causing exon 3 skipping in >90% of mRNA)	Aoyama et al. (2017)
**E3**	c.149del	p.Thr50Asnfs*7	Fukao et al. (1998), (2001), Fukao, Zhang, et al. (2003), Hori et al. (2015)
**E4**	c.286C>T	p.Gln96*	Sarafoglou et al. (2011)
**I4**	c.334+1G>A	splice donor site (probably exon 4 skipping)	Grünert et al. (2017)
**E5**	c.354_355delinsG	p.Cys119Valfs*4	Law et al. (2015)
**E5**	c.414_415del	p.Leu140Tyrfs*36	Paquay et al. (2017)
**I5**	c.435+1G>A	splice donor site (probably exon 5 skipping)	Fukao et al. (1997)
**E6**	c.446del	p.Val149Glyfs*14	Paquay et al. (2017)
**E6**	c.462_482delinsTCCTC	p.Glu154Aspfs*4	Grünert et al. (2017)
**E7**	c.622C>T	p.Arg208*	Fukao, Nguyen, et al. (2010), Sarafoglou et al. (2011), Wen et al. (2016), Nguyen et al. (2015), (2017), Grünert et al. (2017)
**I7**	c.730+1G>A	Splice donor site (probably exon 7 skipping)	Abdelkreem, Akella, et al. (2017)
**I7‐E8**	c.731–46_752del	Splice acceptor site (causing exon 8 skipping)	Fukao, Song, et al. (1995), Fukao, Yamaguchi, et al. (1995), (2001)
**E8**	c.754_755insCT	p.Glu252Alafs*17	Fukao et al. (1997), (2001)
**E8**	c.814C>T	p.Gln272* (75% of mRNA), affects ESE sequence causing exon 8 skipping (25% of mRNA)	Fukao et al. (1994), Sakurai et al. (2007), Paquay et al. (2017)
**I8**	c.826+1G>T	Splice donor site (causing exon 8 skipping)	Fukao, Yamaguchi, Orii, Schutgens, et al. (1992), (2001), Wakazono et al. (1995), Zhang et al. (2004), Paquay et al. (2017), Grünert et al. (2017)
**I8**	c.826+5G>T	Splice donor site (causing exon 8 skipping)	Thümmler et al. (2010)
**I8**	c.826+5_826+9del	Splice donor site (probably exon 8 skipping)	Grünert et al. (2017)
**I9**	c.940+1G>T	Splice donor site (probably exon 9 skipping)	Grünert et al. (2017)
**I9**	c.941–9T>A	Splice acceptor site (causing exon 10 skipping in 90% of transcripts)	Sasai et al. (2017)
**E10**	c.951C>T	Affects ESE sequence causing exon 10 skipping (≈ 40% of mRNA)	Fukao, Horikawa, et al. (2010), Otsuka et al. (2016)
		p.317Asp = (≈ 60% of mRNA)	
**I10**	c.1006–2A>C	Splice acceptor site (causing exon 11 skipping)	Fukao, Yamaguchi, Orii, Schutgens, et al. (1992), (2001), Wojcik et al. (2018)
**I10**	c.1006–1G>C	Splice acceptor site (causing exon 11 skipping)	Fukao, Yamaguchi, Orii, Osumi, et al. (1992), (2001), Nguyen et al. (2015), (2017), Su et al. (2017), Wojcik et al. (2018)
**I10**	c.1006–1G>A	Splice acceptor site (probably exon 11 skipping)	Law et al. (2015)
**E11**	c.1013_1016dup	p.Asp339Glufs*17	Abdelkreem, Akella, et al. (2017)
**E11**	c.1032dup	p.Glu345Argfs*10	Nguyen et al. (2015), (2017)
**E11**	c.1033_1034del	p.Glu345Argfs*9	Paquay et al. (2017)
**E11**	c.1083dup	p.Ala362Serfs*4	Sewell et al. (1998), Fukao et al. (2001)
**I11**	c.1163+2T>C	Splice donor site (activates cryptic splice site causing c.1163_1164ins GCAG)	Fukao et al. (1993), (2001), Grünert et al. (2017)
**E12**	c.1223_1226dup	p.Ala410Serfs*51	Paquay et al. (2017)
	g.20623_29833delinsGTAA	Probably del exons 6–11	Nguyen et al. (2017)
	c.(120+1_121‐1)_(344+1_345‐1)del	del exons 3–4	Fukao et al. (2013)
	c.(72+1_73‐1)_(344+1_345‐1)del, c.(72+1_73‐1)_(435+1_436‐1)del	del exons 2–4 (≈ 10% of mRNA), del exons 2–5 (≈ 90% of mRNA)	Zhang et al. (2006)
	c.(730+1_731‐1)_(940+1_941‐1)dup	Tandem duplication of exons 8–9	Fukao et al. (2007)

Abbreviations: E, exon; ESE, exonic splicing enhancer; I, intron; T2, mitochondrial acetoacetyl‐CoA thiolase

^a^ Description of nucleotide changes, exons/introns, and predicted amino acid change follows the HGVS nomenclature (version 15.11, http://varnomen.hgvs.org; den Dunnen et al., 2016) using *ACAT1* NCBI reference sequences (NM_000019.3, NG_009888.1, and NP_000010.1) with +1 as the number of the A of the ATG initiation codon.

## 3 STRUCTURAL FEATURES OF THE T2 THIOLASE

**Figure 5:**
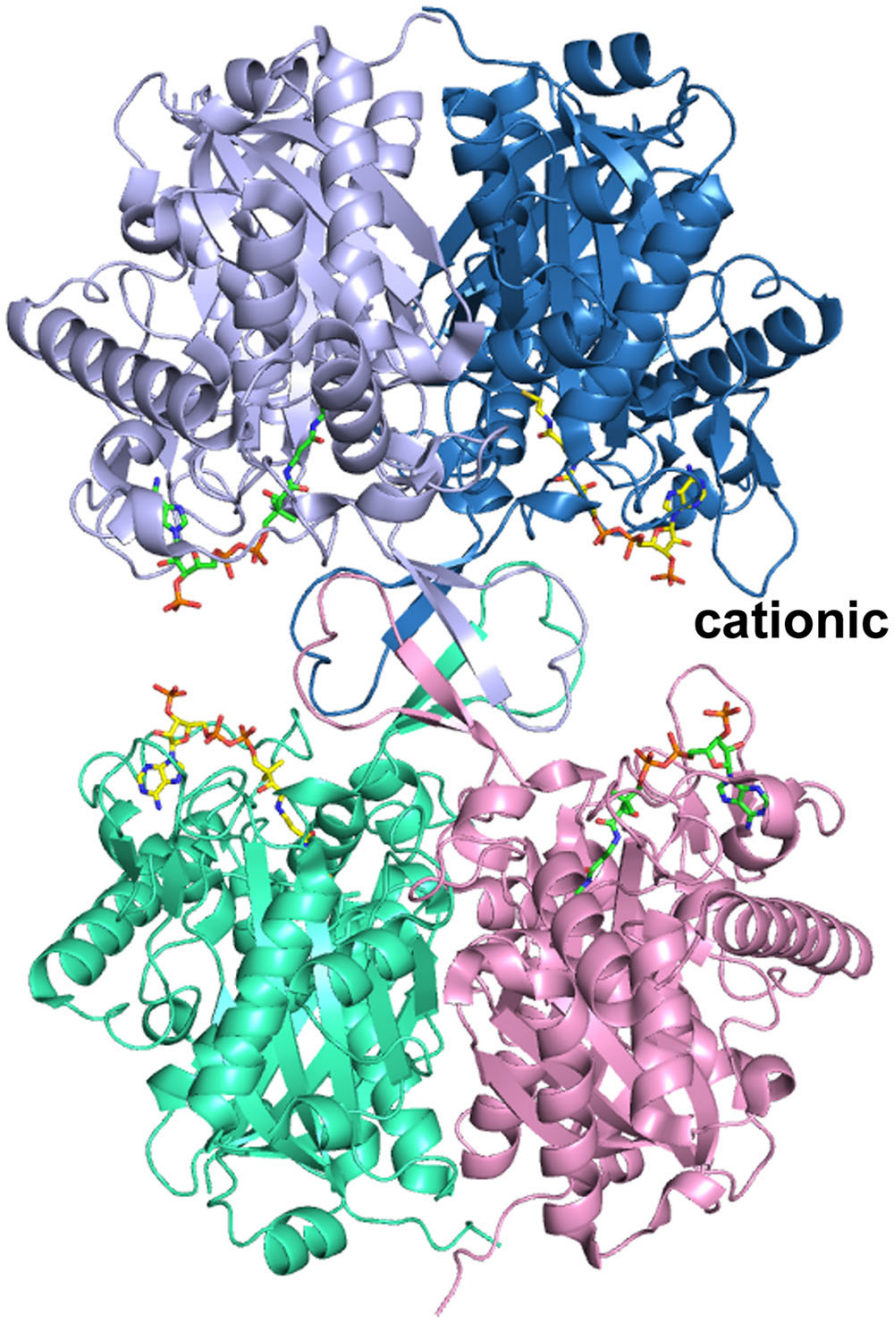
The structure of the T2 tetramer (PDB entry 2IBW), complexed with CoA. The bound CoA molecules are shown as stick models. The two tight dimers (below and above; side view) are assembled into tetramers via the four tetramerization loops (in the middle). “cationic” labels one of the cationic loops, which points to the 3′‐phosphate of the CoA bound in the active site of the opposing dimer. Stereo view is provided in Figure S1

**Figure 6:**
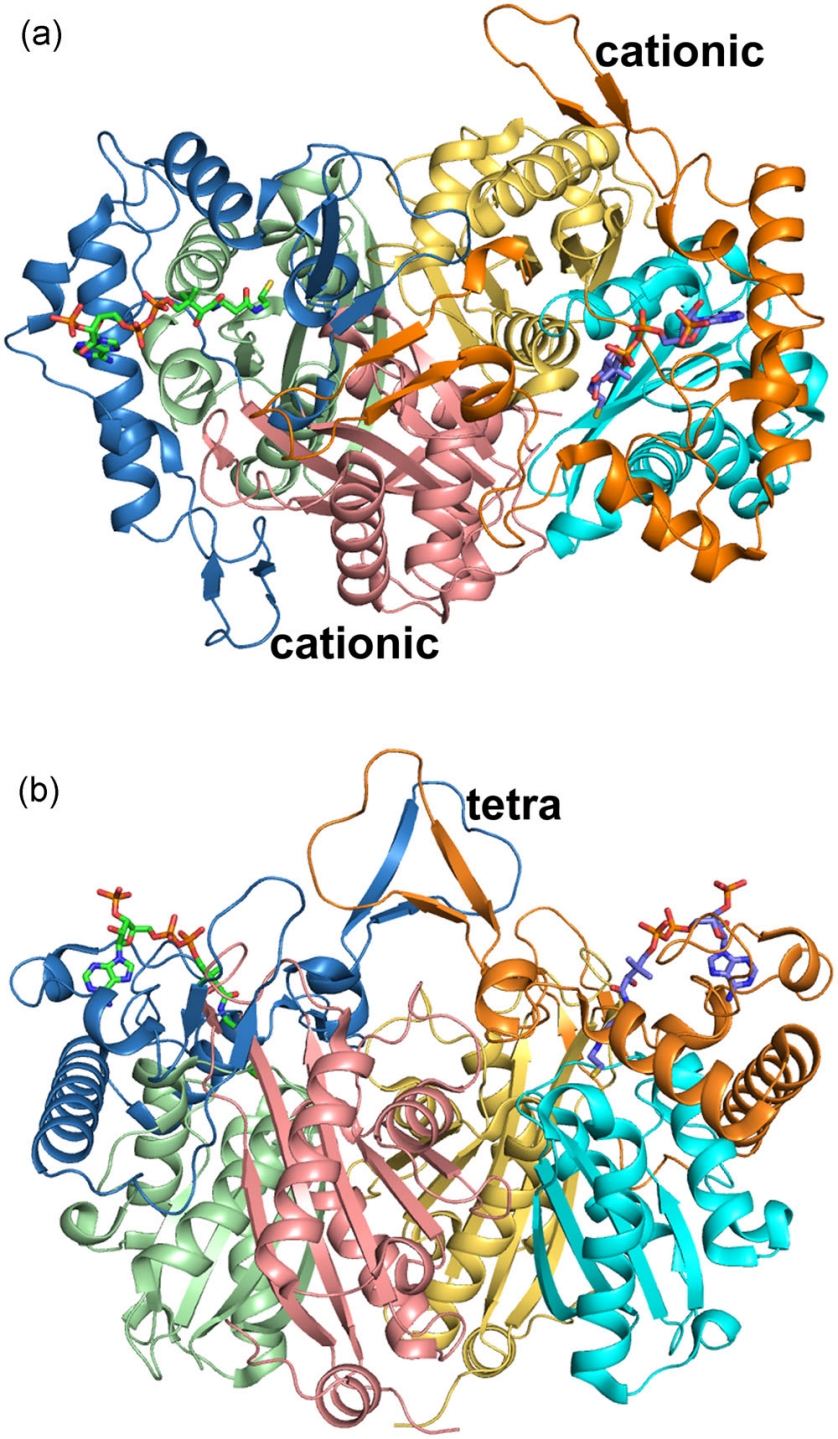
The structure of the T2 tight dimer (PDB entry 2IBW). (a) Top view (view approximately down the local two fold axis of the tight dimer). (b) Side view (rotated by 90° around the horizontal with respect to the top view, same view as in Figure 5). The bound CoA molecules are shown as stick models. In the left subunit, the *N*‐domain, loop domain, and *C*‐domain are colored as purple, blue, and green ribbons, respectively. In the right subunit, the *N*‐domain, loop domain, and *C*‐domain are colored as yellow, orange, and cyan ribbons, respectively. “cationic” and “tetra” identify the cationic and tetramerization loops, respectively. Stereo views are provided in Figure S2

**Figure 7:**
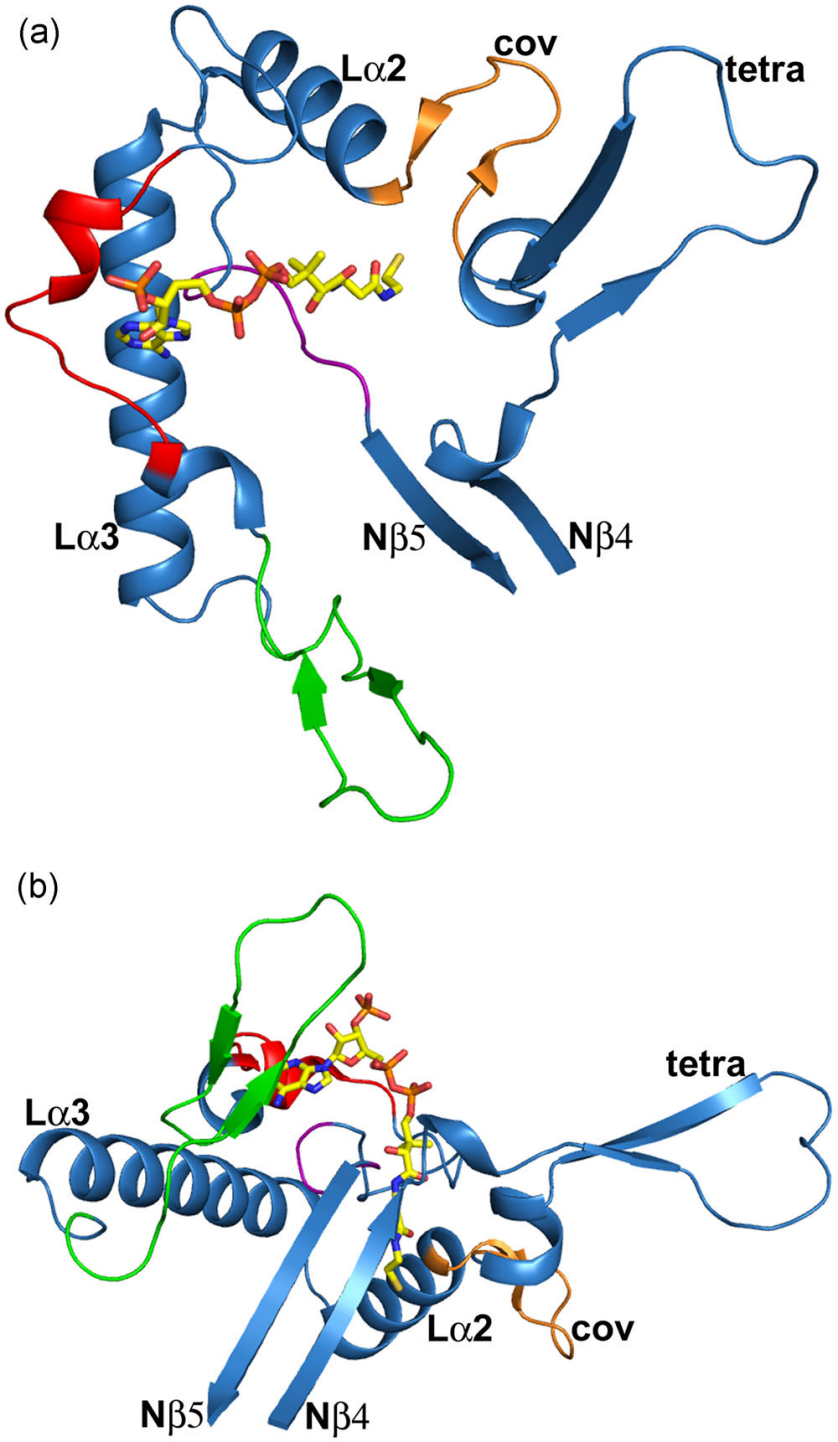
The structure of the T2 loop domain (residues 156–286; PDB entry 2IBW). (a) Top view (same as Figure 6a). (b) Side view (same as Figure 6b). The loop domain protrudes out of Nβ4 and ends at Nβ5 of the *N*‐terminal domain (Figure 3). The covering loop “cov” is in orange, the cationic loop is in green, the adenine loop is in red and the pantetheine loop is in purple. “tetra” identifies the tetramerization loop. The Lα2 and Lα3 helices are also labeled. The bound CoA molecule is shown as a stick model. Stereo views are provided in Figure S3

Human T2 is initially synthesized in the cytosol as a 45‐kDa precursor that matures, following mitochondrial entry, to a homotetramer of 41‐kDa subunits (Fukao et al., 1990; Middleton, 1973). The leader peptide is cleaved off on entry into the mitochondria. The overall structure of the tetramer is shown in Figure 5 and Figure S1. The active site is located at the interface of the tight dimer, as shown in Figure 6 and Figure S2. The construct used for the protein crystallographic studies starts at residue Val34 (which was changed into an alanine to provide better yields when expressed as a recombinant protein in *Escherichia coli*) and the *C*‐terminus is residue Leu427. In the crystal structure (PDB code 2IBW), residues Pro37 to Leu427 are well ordered and are included in the final model (Haapalainen et al., 2007) for each of the four chains of the tetramer. The *N*‐ and *C*‐terminal residues are far away from the catalytic site, being on the opposite site of the subunit. The built model of each subunit has the distinct conserved thiolase superfamily fold that can be subdivided into the *N*‐terminal domain, loop domain, and *C*‐terminal domain (Haapalainen et al., 2007; Kiema et al., 2014, 2019). The *N*‐ and *C*‐terminal domains have the same βαβαβαββ‐topology and these two domains jointly form a five‐layered α‐β‐α‐β‐α structure. The central α‐layer consists of the two active site helices: Nα3 of the *N*‐terminal domain and Cα3 of the *C*‐terminal domain. The structure of the *N*‐terminal domain of T2 is made by residues Pro37–Ser155 and Asn287–Leu309, whereas the *C*‐terminal domain is formed by residues Ala310–Leu427. The loop domain (Figure 7, Figure S3), which is formed by residues Met156–Leu286, covers the closely associated *N*‐ and *C*‐terminal domains of the subunit. The loop domain has two protruding loops: the tetramerization loop (Pro160–Asp177), which stabilizes the tetrameric structure of the enzyme, and the cationic loop (Val232–Asp253). The tip of the cationic loop has a basic side chain (Lys243), pointing toward the active site of the opposing dimer. The cationic loop is possibly important for binding and/or release of the negatively charged CoA substrate at the active site of the opposing dimer (Haapalainen et al., 2007).

The functional site of each of the subunits of the T2 tetramer is very extensive, including not only the catalytic residues but also the residues that shape the CoA binding site. These residues are part of different loops from the core domains (the catalytic loops, Figures 2 and 3) as well as from the loop domain (the CoA‐binding loops, Figures 7 and 3), In addition, the active site of each subunit is near the dimer interface and is therefore fully functional only once the two monomers are assembled correctly at the dimer interface (Figure 6) and also only when loops of each of the two other subunits of the tetramer complete their shape and geometry (Figure 5) (Janardan, Harijan, Kiema, Wierenga, & Murthy, 2015; Kiema et al., 2019). The CoA molecule contains three parts, being the 3ʹ‐phosphate adenosine, pyrophosphate, and pantetheine moieties. The adenine ring of CoA is bound in a small cleft, which is lined by the side chains of Tyr219, Arg258, Val259, Asp260, Lys263, Val264, Leu267, Ala280, and Ala281. The residues Val259 to Leu267 form the adenine binding loop (Figure 7). There is a hydrogen bond interaction between OH(Tyr219) and the amino group of the adenine ring of CoA. In addition, there is the main chain to adenine hydrogen bonds with the adenine loop residues, namely Arg258, Val259, and Asp260. Lys263 forms the only salt bridge between T2 and the 3ʹ‐phosphate moiety of CoA. The pantetheine moiety binds in a narrow tunnel lined by several parts of the loop domain, including the *N*‐terminal end of Lα2 (residues Met193–Thr200), the Cβ2‐Cα2‐loop (residues Asn353–Phe356), and the pantetheine‐loop (Ala280–Thr285; Figure 7). The binding mode of the pantetheine moiety is conserved among different thiolases (Haapalainen et al., 2007).

Four essential catalytic residues project into the catalytic cavity from the four catalytic loops: Cys126, Asn353, His385, and Cys413. Cys126 is the nucleophilic cysteine (Figure 1) and Cys413 is the acid‐base cysteine. These four residues are part of the four respective sequence fingerprints of these loops (Figure 2), being CxS (Nβ3‐Nα3 loop), NEAF (the Cβ2‐Cα2 loop), GHP (Cβ3‐Cα3 loop), and CxG (of the Cβ4‐Cβ5 loop). The Cβ4‐Cβ5 loop is covered by the covering loop (from residues Leu184 to Met193), which is after the tetramerization loop and before Lα2. The catalytic site is (a) completely shielded from bulk solvent and only reachable via the pantetheine binding tunnel and (b) is narrow being able to accommodate the acetoacetate or 2‐methylacetoacetate moieties of acyl‐CoA, but not larger acyl moieties (Haapalainen et al., 2007).

Other important structural features of the catalytic cavity are oxyanion hole 1 (OAH1), which is formed by NE2(His385) and a catalytic water (Wat98, anchored to Asn353) and oxyanion hole 2 (OAH2), which is formed by N(Cys126) and N(Gly415) (Figure 2). Residues Phe325–Pro326 (of the DFP‐loop, which is part of the Cβ1‐Cα1‐loop; Figure 3) provide the binding cavity for the 2‐methyl moiety of the 2‐methylacetoacetyl‐CoA substrate. T2 contains a unique potassium ion binding site, which does not exist in other thiolase subfamilies. Residues with atoms that co‐ordinate the bound potassium ion are located in the loop domain, being Tyr219 of the Lα3‐helix and residues Ala280, Ala281, Ala283, and Thr285 of the pantetheine loop. The potassium ion stabilizes the structure of the pantetheine binding loop. T2 also has a unique chloride ion binding site. The chloride ion is bound near the catalytic site at the dimer interface and it stabilizes the conformation of the Cβ4−Cβ5 loop (Haapalainen et al., 2007).

## 4 DISEASE‐ASSOCIATED *ACAT1* VARIANTS

To date, 105 *ACAT1* variants associated with T2 deficiency have been reported in 149 patients from 134 nuclear families (a family group that consists only of parents and children, Tables 1, 2, 3, Figure 4; Supporting Information Table). Homozygotes are found in 68 (50.8%) families. Data of *ACAT1* genetic analysis are not available for 10 other T2‐deficient patients whose diagnoses were based on T2 enzyme assay of their fibroblasts. We assembled these data through comprehensive literature review (our laboratory work at Gifu University contributed to a lot of these publications); included in these data are two novel *ACAT1* variants and a new case with T2 deficiency described for the first time here. References for disease‐associated *ACAT1* variants are provided in Tables 1, 2, 3, and those for T2‐deficient patients are provided in Supporting Information Table. Description of variants follows the HGVS nomenclature (version 15.11, http://varnomen.hgvs.org; den Dunnen et al., 2016) using *ACAT1* NCBI reference sequences (NM_000019.3, NG_009888.1, and NP_000010.1). We verified compliance with HGVS nomenclature using Mutalyzer program (https://mutalyzer.nl/; Wildeman, van Ophuizen, den Dunnen, & Taschner, 2008). All disease‐associated *ACAT1* variants described herein are submitted to ClinVar public database (https://www.ncbi.nlm.nih.gov/clinvar/).

Disease‐associated *ACAT1* variants are found in every exon (Figure 4). Exon 11 contains the highest number (*n* = 15), followed by exon 6 (*n* = 11), exon 5 (*n* = 9), and exons 7 and 9 (each = 8). As observed in most autosomal recessive diseases, missense *ACAT1* variants are the most common type (*n* = 56). In addition, there are 23 deletions/insertions/duplications. Among the point variants, 21 are associated with aberrant splicing (one of which is a synonymous variant), five are nonsense, and three affect the ATG initiation codon. Of note, certain variants are classified under two categories (e.g., c.949G>A results in both aberrant splicing and the p.Asp317Asn missense variant; Table 1).

Most disease‐associated *ACAT1* variants are “private”, being observed in only one family. Seventy‐six (72.4%) variants have been detected only in single T2‐deficient families, 24 have been identified in between two and four families, and only five variants have been found in five or more families (Supporting Information Table)**.** c.622C>T (p.Arg208*) is the most frequent variant. It has been detected in 28 families, most of which are Vietnamese. This variant accounts for 66% of all *ACAT1* variant alleles identified in Vietnamese patients with T2 deficiency (Nguyen et al., 2017). Recent evidence indicates that the c.622C>T variant has been introduced by an ancient common founder to Vietnamese Kinh ethnic population 1900–2500 years ago (Nguyen et al., 2017). This highly conserved residue is changed into glutamine (c.623G>A, p.Arg208Gln) in two other families (Sakurai et al., 2007). The importance of this residue for the enzymatic function is discussed in subsequent sections.

The second most common disease‐associated *ACAT1* variant is c.1006–1G>C that has been identified in 13 families, most of which are Vietnamese. It affects a highly conserved point at the splice acceptor site of intron 10, altering the Shapiro and Senapathy score from 67 to 49.5 (Shapiro & Senapathy, 1987). cDNA analysis of T2‐deficient patient's fibroblasts revealed that c.1006–1G>C is associated with exon 11 skipping (Fukao, Yamaguchi, Orii, Osumi, & Hashimoto, 1992). Exon 11 skipping causes a frameshift of the coding sequence, which is predicted to exert drastic effects on the variant T2 protein, truncating it prematurely with loss of 53 *C*‐terminal residues. Indeed, the T2 activity and protein were virtually absent in fibroblasts of a patient homozygous for c.1006–1G>C variant (Fukao, Yamaguchi, Orii, Osumi, et al., 1992). The third most common disease‐associated *ACAT1* variant and the most common missense variant is c.578T>G (p.Met193Arg) that has been detected in eight families, most of which are from India. This is followed by c.455G>C (p.Gly152Ala) that was found in six families. Transient expression analysis of both p.Met193Arg and p.Gly152Ala variant T2 cDNAs revealed no residual enzyme activity (Abdelkreem, Akella, et al., 2017; Zhang et al., 2004).

### 4.1 Disease‐associated missense *ACAT1* variants

The disease‐associated missense *ACAT1* variants (*n* = 56) are not uniformly distributed across the *ACAT1* gene (Figure 4). Exons 6 and 11 contain the highest number of such variants (nine for each), followed by exon 9 (eight variants), then exons 5 and 7 (seven for each). The *N*‐terminal part (122 residues, Val34–Ser155), the loop domain (131 residues, Met156–Leu286), and the *C*‐terminal part (141 residues, Asn287–Leu427) contain 13, 22, and 21 variants, respectively (Figure 4).

### 4.2 Structure‐function relationship of missense variants whose expression levels are equal or greater than 25% that of wild‐type

The 30 disease‐associated missense *ACAT1* variants listed in Table 1 concern those variants for which the catalytic and expression properties have been determined at 37°C. All variants listed in Table 1 have low activities; c.431A>C (p.His144Pro) variant has the highest activity (25% that of wild‐type T2). For only two variants, c.377G>C (p.Cys126Ser) and c.655T>C (p.Tyr219His), the expression level is similar to that of wild‐type; however, the catalytic activity for these two variants is 0%. From the structure analysis, it can be seen that both residues are essential for enzyme function. Tyr219 interacts both with the potassium ion and with the adenine moiety of CoA and Cys126 is the nucleophilic cysteine (Figure 1) of the catalytic site.

Apart from p.Cys126Ser and p.Tyr219His, there are six other variants whose expression levels are equal or greater than 25% that of wild‐type: c.278A>G (p.Asn93Ser), p.His144Pro, c.556G>T (p.Asp186Tyr), p.Arg208Gln, c.844A>C (p.Asn282His), and c.968T>C (p.Ile323Thr). The variants p.Asn93Ser (T2 protein level, 60%; measured activity, 8%) and p.His144Pro (T2 protein level, 50%; measured activity, 25%) are near the dimer interface. We speculate that these variants are properly expressed, but that the folded monomers cannot form stable dimers and therefore the catalytic activity of these variants is indeed low. Recent studies of the dimeric zebrafish SCP2‐thiolase (Kiema et al., 2019) show that for this thiolase the folded monomeric form is stable (but predicted to be catalytically inactive). By extension, T2 monomers may also be stable but catalytically inactive. Of the remaining four variants whose expression levels are equal or greater than 25% that of wild‐type, three of them are in the loop domain (Figure 7): p.Asp186Tyr, p.Arg208Gln, and p.Asn282His. Furthermore, the p.Ile323Thr variant is in the Cβ1‐Cα1 loop that interacts with the loop domain. Each of these four residues is in a surface loop. From the structure, it is predicted that these four variants would allow the formation of the tetramers assembly but nevertheless, the catalytic activity is very low for p.Asp186Tyr, p.Arg208Gln, and p.Asn282His. Only p.Ile323Thr has a catalytic activity of 20% that of wild‐type T2. Further information on the structure‐function relationship of these four variants is given below.

**Figure 8:**
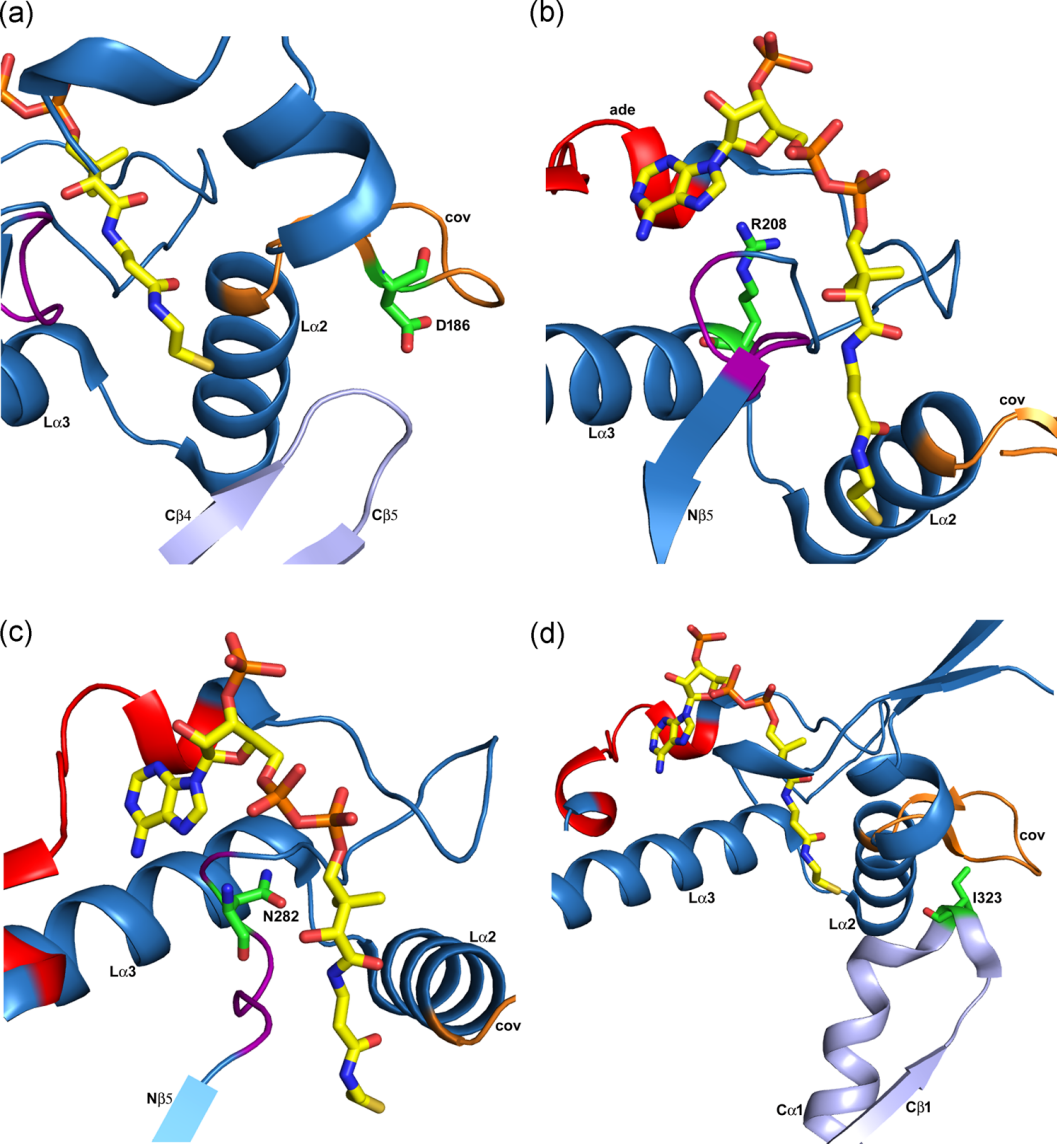
Missense variants of residues in loops on the surface of the T2 tetramer (PDB entry 2IBW). The visualized loop residues are either in the loop domain (panels a, b, c) or interact with the loop domain (panel d). Expression of variant T2 cDNAs containing these variants produces T2 protein levels of 25% or higher compared to wild‐type T2, as discussed in the text. These panels are zoomed‐in views, of the loop domain (same view as in Figure 7b). The covering loop (“cov”) and the Lα2 and Lα3 helices are labeled in each panel. The bound CoA molecule is shown as a stick model. (a) The p.Asp186Tyr variant (T2 protein level, 33%; measured activity, 0%). Asp186 (D186) is in the covering loop (orange) and points to the Cβ4‐Cβ5 loop (light blue) of the catalytic site. (b) The p.Arg208Gln variant (T2 protein level, 50%; measured activity, 0%). Arg208 (R208) is at the beginning of helix Lα3 (cyan). The Arg208 side chain is hydrogen bonded to the loop region just after the adenine loop (“ade”, shown in red). (c) The p.Asn282His variant (T2 protein level, 50%; measured activity, 0%). Asn282 (N282) is in the pantetheine loop (purple). (d) The p.Ile323Thr variant (T2 protein level, 25%; measured activity, 20%). Ile323 (I323) is in the Cβ1‐Cα1 loop (light blue) of the *C*‐terminal domain, just before the DFP sequence fingerprint of the binding pocket for the 2‐methyl group of the 2‐methylacetoacetyl‐CoA substrate. cDNA, complementary DNA

The p.Asp186Tyr variant (T2 protein level, 33%; measured activity, 0%) is in the covering loop (Figure 8). This loop stabilizes the conformation of the Cβ1‐Cα1 loop and the Cβ4‐Cβ5 loop. The latter loop provides the acid/base cysteine, Cys413, which is an essential catalytic residue and this loop also contributes to OAH2. These two functionalities are essential for the catalytic properties and therefore it is predicted that this variant inactivates T2 thiolase.

The p.Arg208Gln variant (T2 protein level, 50%; measured activity, 0%) is in Lα3. The Arg208 side chain makes a hydrogen bond interaction with OD2(Asp212) and the backbone oxygen of Leu267, which is in a loop region immediately after the adenine binding loop. The latter interaction stabilizes this loop at the correct position for substrate binding (Figure 8).

The p.Asn282His variant (T2 protein level, 50%; measured activity, 0%) is in the pantetheine loop, at the end of the loop domain, shaping the binding pocket of the potassium ion as well as the pantetheine binding tunnel (Figure 8). The variant residue distorts the pantetheine loop and therefore the catalytic function is lost.

The p.Ile323Thr variant (T2 protein level, 25%; measured activity, 20%) is in the Cβ1‐Cα1 loop, which is near the binding pocket of the 2‐methyl group of the substrate (Figure 8). The 20% catalytic activity, as measured with the acetoacetyl‐CoA substrate, suggests that the mature enzyme is fully active. The p.Ile323Thr variant changes Ile323 into a threonine, which is much more polar. Although the Ile323 side chain points into bulk solvent, we speculate that this variant changes the conformation of the Cβ1‐Cα1 loop, disrupting the binding pocket of the 2‐methyl group of the 2‐methylacetoacetyl‐CoA substrate, possibly without disrupting the degradation of acetoacetyl‐CoA.

### 4.3 Structural analysis of all missense variants

For each of the missense variants listed in Table 1, information is provided in the last column concerning the location of the variant residue with respect to the structure of T2. Considering the structure of the tetramer, three residue categories have been defined, being either (a) completely buried (“buried”), or (b) near the surface, being partially buried (“surface”) or (c) having a side chain that points towards the solvent (“exposed side chain”). For some of the latter residues, the side chain is interacting closely with the rest of the protein. If the solvent exposed side chain is only loosely interacting with the rest of the protein, then for variants which do not change much the side chain properties (e.g., no change in polarity), it is predicted that the in vivo folding efficiency or stability will be similar to that of wild‐type. This simple prediction scheme is not valid whenever the variant concerns a proline and/or glycine. In Table 1, it concerns seven out of the 30 listed variants, which are either buried or surface residues. Except for p.His144Pro (T2 protein level, 50%; measured activity, 25%), these variants are poorly expressed (protein level is equal or less than 10%).

Variants that are predicted not to change the folding or stability properties, but nevertheless are observed to be disease‐associated, identify residues that are important for the catalytic properties of the native tetramer assembly. The current set of disease‐associated variants listed in Table 1 includes five such residues, Cys126, Met193, Arg208, Tyr219, and Ile323. The p.Cys126Ser and p.Tyr219His variants are both expressed at the same level as that of wild‐type, as discussed in the previous section. The p.Arg208Gln and the p.Ile323Thr variants have also been discussed in the previous section. The p.Met193Arg variant concerns a residue whose side chain points toward bulk solvent and is therefore predicted not to interfere with folding or stability. However, the side chain of Met193 points into the narrow pantetheine binding tunnel. The experimental data show that the bulkier and more polar arginine side chain does not allow proper folding of this T2 thiolase variant.

Most of the residues listed in Table 1 (16 out of 30) are completely buried in the structure. These variants are predicted to adversely affect the folding and/or stability and therefore are predicted to have lower catalytic efficiency. Indeed, the expression levels of all these variants are less than 25% that of wild‐type, except for p.Asn93Ser, p.Asp186Tyr, and p.Asn282His (Figure 8). The latter two variants are located in loops near the surface of the tetramer, which apparently allows for partial folding, but the mature protein has low catalytic activity, as discussed above. Asn93 is buried at the dimer interface and the variant residue is predicted to prevent assembly of the functional tetramer, as also discussed above.

Table 2 also lists missense disease‐associated variants, but for these variants, there are no folding/stability or activity data. These variants have also been mapped onto the structure, and also for this set, most of the variants (23 out of 26) concern residues that are completely or partially buried. These 23 variants are predicted to produce expression levels less than that of wild‐type. Some of these variants concern residues that are located at the monomer‐monomer interface of the tight dimer. It is possible that these variants may be expressed in a soluble, monomeric form, but these forms are likely not active.

It will be particularly interesting to find out the expression and activity properties of the variants of Table 2, which are classified as having “exposed side chain”. It concerns the three variants c.578T>C (p.Met193Thr), c.760G>A (p.Glu254Lys), and c.1160T>C (p.Ile387Thr). The p.Met193Thr variant concerns a residue which is located at the beginning of helix Lα2, and whose side chain is usually a hydrophobic residue, pointing into the narrow pantetheine binding tunnel. Like for the p.Met193Arg variant (Table 1), the p.Met193Thr variant replaces a hydrophobic side chain by a polar side chain, which is then predicted to interfere with the binding of the pantetheine moiety in its tunnel and therefore interferes with the catalytic properties. The p.Glu254Lys variant concerns a residue of which the side chain is hydrogen bonded to the rest of the protein. It is located far away from the catalytic site, being in a surface loop just after the cationic loop (Figure 7). The function of the cationic loop has not been studied experimentally, but the structure of the tetramer suggests that the cationic side chain at the tip of the loop (Lys243) might be important for the efficient capture and/or release of the substrate. The p.Glu254Lys variant could allow proper folding and/or stability of the T2 tetramer but it might subsequently interfere with the functional properties of the cationic loop and therefore causes loss of optimal function. The p.Ile387Thr variant is near the active site, being at the beginning of the Cα3‐helix. It points into the catalytic site and is therefore predicted to interfere with proper catalytic function.

### 4.4 Disease‐associated splice variants

Most aberrant splicing‐associated variants affect splice donor (*n* = 9) and acceptor (*n* = 6) sites in the *ACAT1* gene (Figure 4; Table 3). These variants generally reside at the highly conserved sequences: last nucleotide of exon, position +1/+2/+5 at the splice donor site, and position −1/−2 at the splice acceptor site. Nevertheless, two of these variants, c.121–13T>A and c.941–9T>A, are located at the polypyrimidine tract of the splice acceptor site. Although in silico tools failed to predict the pathogenic effect of the latter two variants on splicing, minigene splicing experiments recently proved that c.121–13T>A and c.941–9T>A variants induce skipping of exons 3 and 10, respectively, in greater than 90% of transcripts (Aoyama et al., 2017; Sasai et al., 2017).

On the other hand, there are five exonic variants that cause aberrant splicing through one of two mechanisms. First, an exonic variant activates a cryptic splice site within an exon. c.380C>T (p.Ala127Val) activates a cryptic splice acceptor site located five bases downstream from the c.380 position in exon 5. This causes aberrant splicing with a c.336–386 (51 bp) deletion in approximately 94% of transcripts, removing the catalytically essential residue Cys126 (Nakamura et al., 2001). Likewise, c.1124A>G (p.Asn375Ser) activates a cryptic splice donor site located five bases upstream from the c.1124 position in exon 11. This results in a c.1120–1163 (44 bp) deletion in approximately 89% of the T2 messenger RNA (mRNA). The resulting frameshift replaces the last 54 amino acid residues, including the catalytically essential residues His385 and Cys413, with 69 different *C*‐terminal residues (Fukao, Boneh, Aoki, & Kondo, 2008). In the second mechanism, an exonic variant alters the consensus sequence of an exonic splicing enhancer (ESE) site. c.814C>T (p.Gln272*) is located within an ESE sequence for SRSF2 (c.^808^GTTTTCCA; nucleotides number 78–85 of exon 8), which is a potential binding site for serine/arginine‐rich splicing factor 2 (ESE finder version 3.0; http://krainer01.cshl.edu/cgi-bin/tools/ESE3/esefinder.cgi?process=home; Cartegni, Wang, Zhu, Zhang, & Krainer, 2003). In fact, a minigene splicing experiment demonstrated that c.814C>T results in skipping of exon 8 in 25% of transcripts (Fukao et al., 1994). Likewise, both c.949G>A (p.Asp317Asn) and c.951C>T (p.=) are located in an ESE sequence for SF2/ASF (c.^947^CTGACGC; nucleotides number 7–13 of exon 10). Minigene splicing experiments also demonstrated that c.949G>A and c.951C>T variants cause skipping of exon 10 in 80% and 40% of transcripts, respectively (Fukao, Horikawa, et al., 2010, Otsuka et al., 2016). Without this information, c.951C>T might wrongly be regarded only as a benign synonymous variant. Of note, RNA sequencing is a useful technology to reveal abnormally spliced transcripts.

### 4.5 Disease‐associated deletion/insertion/duplication variants

These variants are also listed in Table 3. It is difficult to predict how such variants would affect the folding and/or stability of the T2 protein. In such cases, expression analysis of variant cDNAs has not been routinely performed.

Furthermore, five large deletions/insertions/duplications have been reported in the *ACAT1* gene. g.20623_29833delinsGTAA includes deletion of exons 6–11 (Nguyen et al., 2017). c.731–46_752del (a 68‐bp deletion) involves the splice acceptor site of intron 7, causing exon 8 skipping (Fukao, Song, et al., 1995). The other three variants could be attributed to *Alu* elements‐mediated unequal homologous recombination (Fukao et al., 2007, 2013; Zhang et al., 2006). We established multiplex ligation‐dependent probe amplification (MLPA) analysis for *ACAT1*, which is useful to identify these large gene rearrangements (Fukao et al., 2013). Of note, a recent *ACAT1* minigene experiment demonstrated that insertion of *Alu*Y–partial *Alu*Sz6–*Alu*Sx in an antisense direction within intron 9 has a negative effect on exon 10 inclusion. This effect is (a) distance dependent—the shorter the distance between the antisense *Alu* element and exon 10, the greater the skipping of exon 10; (b) additive with that of an ESE variant (c.951C>T) in exon 10; and (c) canceled by the c.941C>G substitution at the first nucleotide of exon 10, which optimizes the splice acceptor site of intron 9. Accordingly, intronic antisense *Alu* elements have a negative splicing effect on close downstream exons, particularly when splice acceptor sites are suboptimal (Nakama et al., 2018).

### 4.6 Other disease‐associated variants

Five nonsense *ACAT1* variants have been reported (Table 3; Figure 4). mRNAs with premature termination are mostly subjected to nonsense‐mediated decay. In the case of c.814C>T (p.Gln272*), this also causes skipping of exon 8 in 25% of transcripts. This phenomenon was previously termed as nonsense‐associated alternative splicing (Fukao et al., 1994) and recently designed as exon skipping caused by a variant at an ESE sequence (discussed above). On the other hand, c.1A>G, c.2T>A, and c.2T>C variants affect the ATG initiation codon with reduced translation efficiency of 11, 7, and 22%, respectively (Fukao, Matsuo, et al., 2003).

### 4.7 Biochemical and laboratory significance

The genotype exerts a considerable effect on the biochemical phenotype of patients with T2 deficiency. Based on the T2 enzymatic activity detected on expression of variant cDNAs, patients with T2 deficiency can be divided into two categories: Those with “mild” variants, in whom at last one of the two variant alleles retains some residual T2 activity, and those with “severe” variants, in whom none of the two variant alleles has any residual T2 activity (Fukao et al., 2001). Patients with mild variants can develop episodic ketoacidosis as severe and frequent as those with severe variants; however, the above mentioned isoleucine‐catabolic intermediates, essentially TIG, tend to be more subtle in the former patients not only in stable states but also during acute ketoacidosis (Fukao et al., 2001, Fukao, Zhang, et al., 2003, Fukao et al., 2012). This has important practical implications. First, critical samples (serum, blood, urine) have to be collected from acutely presented patients at once before starting treatment. Second, subtle/atypical abnormalities in urinary organic acids or blood acylcarnitine analyses do not absolutely exclude a diagnosis of T2 deficiency in patients with a consistent presentation. Third, newborn screening by tandem mass spectrometry may miss more T2‐deficient patients in regions where mild variants predominate, such as in Japan, rather than in regions with a preponderance of severe variants, such as in Vietnam. In general, a normal newborn screening result does not absolutely exclude a diagnosis of T2 deficiency (Abdelkreem et al., 2016; Fukao et al., 2012; Sarafoglou et al., 2011).

Diagnosis of T2 deficiency can be confirmed in a suspected patient by the T2 enzyme assay, preferably using fibroblasts, or by detecting biallelic disease‐associated variants in the *ACAT1* gene. This highlights the importance of the characterization of *ACAT1* variants detected in T2‐deficient patients along with available functional studies. On the other hand, conventional sequence analysis of genomic DNA cannot properly identify copy number variants and certain splicing abnormalities. Detection of copy number variants requires other techniques, such as MLPA or real‐time polymerase chain reaction. cDNA and minigene splicing studies are useful for revealing the impact of certain variants on splicing (see above). Identifying disease‐associated *ACAT1* variants does not only confirm the diagnosis of T2 deficiency in the proband but also enables screening of other family members for finding yet asymptomatic patients and for providing proper genetic counseling. Implementing preventive measures for asymptomatic patients could protect them against potentially lethal ketoacidotic episodes. Prenatal diagnosis becomes also applicable; however, it is not necessarily superior to timely postnatal diagnosis, given that T2 deficiency rarely manifests during the neonatal period (Abdelkreem et al., 2016; Fukao et al., 2014).

## 5 CLINICAL SIGNIFICANCE

Patients with T2 deficiency typically manifest between 6 and 18 months of age with episodic ketoacidosis. A history of ketogenic triggers, such as prolonged fasting or febrile illness, is usually present. Severity and frequency of episodes vary among patients. A considerable proportion of patients suffer from severe ketoacidotic episode/s, sometimes with encephalopathy and/or hemodynamic collapse. Death or permanent neurological abnormalities (e.g., gait disturbances, movement disorders, hypotonia, and mental retardation) are well documented potential complications. On the other hand, some patients remain asymptomatic (Abdelkreem et al., 2016; Fukao et al., 2014, 2018). Of note, secondary carnitine deficiency is rare in T2 deficiency but if present, it may suppress β‐oxidation and modify the clinical manifestation of T2 deficiency from ketoacidotic to hypoketotic hypoglycemic events (Alijanpour et al., 2019).

Patients with T2 deficiency had been thought to be asymptomatic between episodes unless a previous severe episode of ketoacidosis causes irreversible neurological damage. However, an increasing body of evidence indicates that chronic neurological impairment, mainly extrapyramidal manifestations, can exist independent of frank ketoacidosis even in patients with T2 deficiency confirmed at the molecular level (Buhaş et al., 2013; Fukao et al., 2018; Paquay et al., 2017). In vitro studies indicate that 2MAA and 2M3HB exert neurotoxic effects (Leipnitz et al., 2010; Rosa et al., 2005).

Given the large number of private (occurring only in one family) disease‐associated *ACAT1* variants, T2 deficiency lacks an obvious correlation between the genotype and the clinical phenotype, including the age at onset, severity and frequency of ketoacidotic episodes, and eventual outcome. This is evident from the provided comprehensive list of patients with T2 deficiency reported in the literature (Supporting Information Table). Environmental/acquired factors, such as ketogenic triggers, considerably contribute to the clinical presentation (Thümmler, Dupont, Acquaviva, Fukao & De Ricaud, 2010). However, several reports show that T2 deficiency has variable clinical phenotypes even among patients who share not only identical genotype but also similar environmental factors (Abdelkreem, Alobaidy, et al., 2017; Fukao et al., 2012; Köse et al., 2016; Nguyen et al., 2017; Thümmler et al., 2010). Proper acute and preventive treatment seems crucial for a favorable outcome (Hori et al., 2015; Nguyen et al., 2017).

## 6 CONCLUDING REMARKS

Functional studies of 30 disease‐associated missense T2 variants have been performed in vitro, using the potassium ion‐activated acetoacetyl‐CoA degradation assay and for all these variants low activity (equal or less than 25% that of wild‐type T2) is observed (Table 1). From the available information, patients with T2 deficiency can be divided into those with “mild” variants, in whom at least one of the two variant alleles retains some residual T2 activity, and those with “severe” variants, in whom none of the two variant alleles has any residual T2 activity. However, patients with mild variants can develop episodic ketoacidosis as severe and frequent as those with severe variants (Fukao et al., 2001). This raises questions whether the T2 activity measured in vitro using acetoacetyl‐CoA as a substrate fully reflects the in vivo T2 deficiency, and whether it is better to use 2‐methylacetoacetyl‐CoA (or both acetoacetyl‐CoA and 2‐methylacetoacetyl‐CoA) as specific substrates. Indeed, 2‐methylacetoacetyl‐CoA thiolase assay is more sensitive for detecting isoleucine catabolism deficiencies (Middleton & Bartlett, 1983). This substrate is not currently commercially available but can be prepared by published protocols. For one disease‐associated variant (p.Ile323Thr; 25% expression, 20% remaining activity) the variant could affect the structure of the loop that shapes the binding pocket of the 2‐methyl group. The experimental data suggest that the mature enzyme variant is fully active, but from the structural analysis it is predicted that the activity for the 2‐methylacetoacetyl‐CoA substrate could be much more affected. In the latter case, the isoleucine catabolism is much more affected than the ketone body metabolism. In any case, the analysis of the structural context of the missense variants shows that they concern also residues that are not near the active site, and it suggests that in almost all cases the identified disease‐associated *ACAT1* variants concern residues that are buried in the mature protein (Table 1) and therefore are predicted to deteriorate the stability and/or folding properties of the respective T2 variants, thereby decreasing the capacity to efficiently degrade 2‐methylacetoacetyl‐CoA as well as acetoacetyl‐CoA.

Several patients with T2 deficiency developed chronic neurological impairment, mainly extrapyramidal, independent of frank ketoacidosis (Buhaş et al., 2013; Fukao et al., 2018; Paquay et al., 2017). In vitro studies indicate that 2MAA and 2M3HB exert neurotoxic effects (Leipnitz et al., 2010; Rosa et al., 2005). Therefore, accumulated isoleucine‐catabolic metabolites may contribute to neurological impairment in patients with T2 deficiency (Fukao et al., 2018; Paquay et al., 2017). Accordingly, T2 deficiency should be considered not only as a ketolytic defect but also as a defect in isoleucine catabolism with the potential for insidious cerebral toxicity. However, it is likely that other genetic or environmental factors contribute to the neurotoxic effect of isoleucine metabolites, explaining why only a minority of T2‐deficient patients have such neurological manifestations independent of the occurrence of severe metabolic crises. This is an important topic for future research. Another unresolved issue is the therapeutic implications; the effectiveness of carnitine supplementation and protein, particularly isoleucine, restriction in preventing chronic neurological impairment remains to be determined (Fukao et al., 2018).

Finally, many reported *ACAT1* variants in patients diagnosed with T2 deficiency lack the experimental proof of decreased T2 activity (Tables 2 and 3). Structural analysis and in silico tools are useful to predict the pathogenic effect, but such predictions are not always true (see Table 1 and Section 4.4). In these cases, further laboratory studies to demonstrate the decreased T2 activity of such variants are required to confirm the diagnosis.

## FUNDING INFORMATION

This research was supported by a Grant‐in‐Aid for Scientific Research from the Ministry of Education, Culture, Sports, Science and Technology of Japan (grant number 16K09962), by AMED under grant number JP17ek0109276, and by Health and Labor Sciences Research Grants (H29‐nanchitou(nan)‐ippan‐051) for research on rare and intractable diseases.

## CONFLICT OF INTERESTS

The authors declare that there are no conflict of interests.

## AUTHOR CONTRIBUTIONS

Toshiyuki Fukao initiated the idea, performed some novel functional experimental studies, and critically supervised and reviewed all steps of this project. Elsayed Abdelkreem reviewed the literature, gathered and analyzed patients’ and variant data, submitted variants to ClinVar public database, performed some functional experimental studies, and wrote the first draft of the manuscript. Rikkert K. Wierenga and Rajesh K. Harijan mapped the diseases‐associated missense *ACAT1* variants on the structure of the human T2 thiolase, carried out their structural analysis, made the figures visualizing this structural information and contributed to the writing of the manuscript. Seiji Yamaguchi has a long‐term implication with the molecular characterization of T2 deficiency, initially supervised Toshiyuki Fukao in this work, and critically read the draft of this manuscript. All authors approved the final manuscript as submitted.

## Supporting information

Supplementary information
