# Supplementary material for: Mutation update on ACAT1 variants associated with mitochondrial acetoacetyl‐CoA thiolase (T2) deficiency
Source: Hum Mutat. 2019 Jul 3;40(10):1641–63. doi: 10.1002/humu.23831 (PMC6790690; doi:10.1002/humu.23831)
Supplement: Supplementary file 1 — Supplementary information [file HUMU-40-1641-s001.pdf]

Mutation update on *ACAT1* variants associated with mitochondrial acetoacetyl-CoA thiolase (T2) deficiency

Elsayed Abdelkreem[1,2], Rajesh K. Harijan[3], Seiji Yamaguchi[4], Rikkert K. Wierenga[5], Toshiyuki Fukao[1]

[1] Department of Pediatrics, Graduate School of Medicine, Gifu University, Gifu, Japan
[2] Department of Pediatrics, Faculty of Medicine, Sohag University, Sohag, Egypt
[3] Department of Biochemistry, Albert Einstein College of Medicine, Bronx, NY 10461, United States.
[4] Department of Pediatrics, Shimane University School of Medicine, Izumo, Japan
[5] Biocenter Oulu and FBMM, University of Oulu, Oulu, Finland

Supplementary Files

Supp. Figure S1. Stereo view of the structure of the T2 tetramer (side view).

Supp. Figure S2. Stereo view of the structure of the T2 tight dimer. A) Top view. B) Side view.

Supp. Figure S3. Stereo view of the structure of the T2 loop domain. A) Top view. B) Side view

Supp. Table. Documented patients with mitochondrial acetoacetyl-CoA thiolase deficiency (n=159).

Supp. Material. Summary of the experimental methods concerning the expression and catalytic assays of the *ACAT1* variants associated with mitochondrial acetoacetyl-CoA thiolase (T2) deficiency, as listed in **Table 1**.

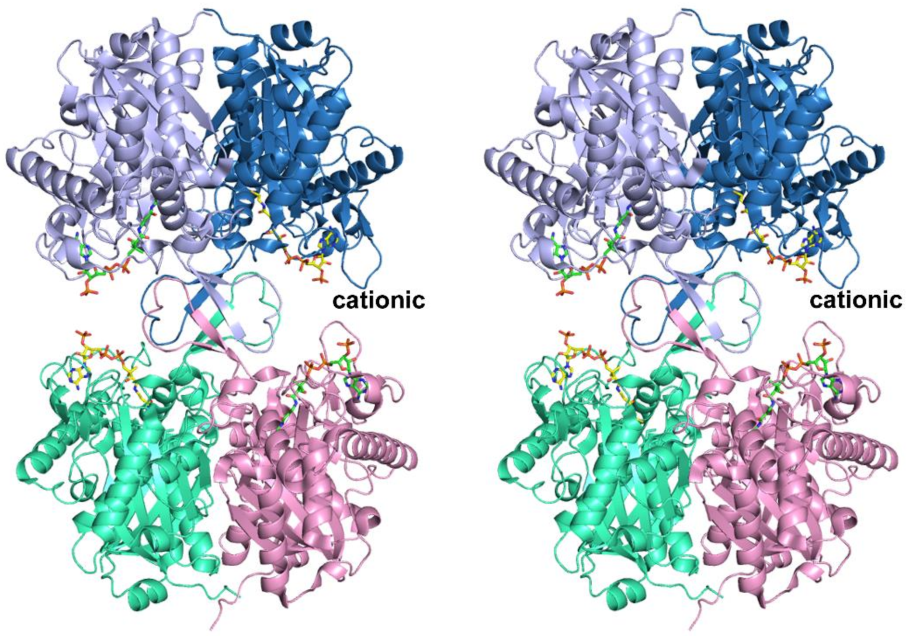

Supp. Figure S1. Stereo view of the structure of the T2 tetramer, complexed with CoA (side view, PDB entry 2IBW).

The bound CoA is shown as sticks. “cationic” labels the cationic loop of the dark-blue subunit. This cationic loop points to the 3’-phosphate of the CoA that is bound in the active site of the opposing purple subunit.

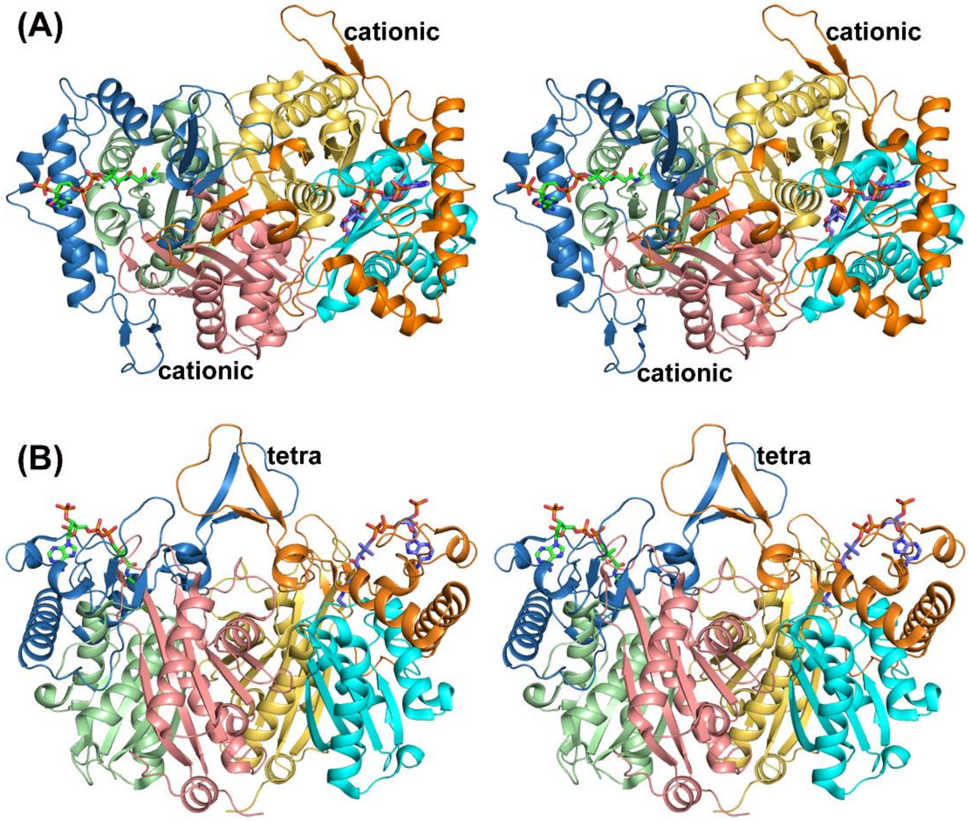

Supp. Figure S2. Stereo view of the structure of the T2 tight dimer (PDB entry 2IBW).

The bound CoA is shown as sticks. A) Top view. B) Side view (same view as in **Supp. Figure S1**). “cationic” identifies the two cationic loops. “tetra” identifies the two tetramerization loops which (in the tetramer, **Supp. Figure S1**) interact with the two tetramerization loops of the second tight dimer to form the tetramer. The loop domain is colored blue in the left subunit and orange in the right subunit.

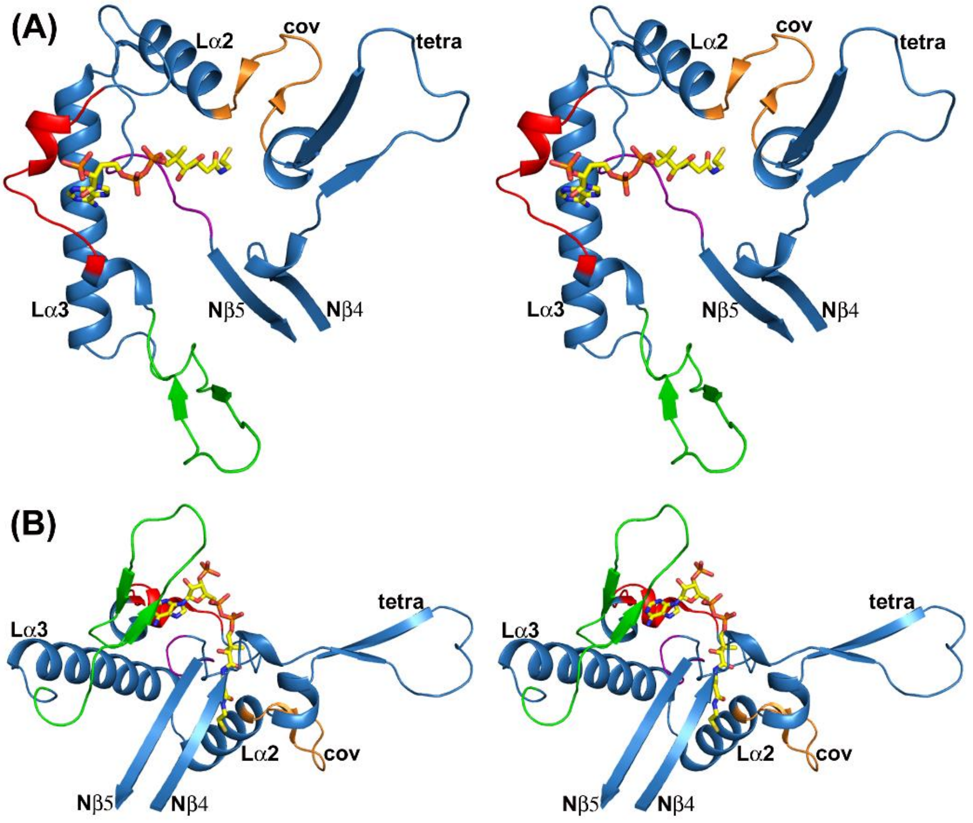

Supp. Figure S3. Stereo view of the structure of the T2 loop domain (PDB entry 2IBW).

The bound CoA is shown as sticks. The loop domain (cyan) corresponds to the loop domain of the left subunit of **Supp. Figure S2**. A) Top view, same as in **Supp. Figure S2A**. B) Side view, same as in **Supp. Figure S2B**. It can be clearly seen that the loop domain is an insertion between the Nβ4 and Nβ5 β-strands of the N-domain. The green loop is the cationic loop. The red loop is the adenine loop. The purple loop is the pantetheine loop. "tetra" identifies the tetramerization loop. "cov", Lα2 and Lα3 label the covering loop, the Lα2 helix and the Lα3 helix, respectively.

Supp. Table. Documented patients with mitochondrial acetoacetyl-CoA thiolase deficiency (n=159).

Patient (ID)[a]	Sex	Race (nationality)	Consa-nguin.	Family history	Age at Onset	Neuro-Develop. outcome	Nucleotide (predicted amino acid) changes[b]	References
1 (GK01)	M	Japanese (Japan)	—	—	20 m	Impaired	c.149del (p.Thr50Asnfs*7) / c.997G>C (p.Ala333Pro)	Hiyama et al (1986), Yamaguchi et al (1988, 1992, 1993), Fukao et al (1990, 1995b, 1998, 2001, 2003a, 2018), Hori et al (2015)
2 (GK02)	F	Spanish (USA)	—	+[c]	16 m	Impaired	c.254_256del (p.Glu85del) / c.455G>C (p.Gly152Ala)	Middleton et al (1986), Nagasawa et al (1989), Fukao et al (1990, 1995b, 2002)
3 (GK03)	M	Laotian (USA)	—	+[c]	10 m	Favorable	c.941-9T>A (splice acceptor site; causing exon 10 skipping in 90% of transcripts) homozygous	Middleton et al (1986), Nagasawa et al (1989), Fukao et al (1990, 1995b), Sasai et al (2017)
4 (GK04)	M	Caucasian (Netherland)	—	+[d]	4 y	Favorable	c.547G>A (p.Gly183Arg) / c.1006-2A>C (splice acceptor site; causing exon 11 skipping)	Schutgens et al (1982), Nagasawa et al (1989), Yamaguchi et al (1993), Fukao et al (1990, 1992a, 1995b, 2018)
5 (GK05)	M	Caucasian (Netherland)	—	+[d]	—	Favorable	c.547G>A (p.Gly183Arg) / c.826+1G>T (splice donor site; causing exon 8 skipping)	Schutgens et al (1982), Nagasawa et al (1989), Yamaguchi et al (1993), Fukao et al (1992a, 1995b, 2018)
6 (GK06)	M	Caucasian (Germany)	—	—	6 m	Impaired	c.1138G>A (p.Ala380Thr) / no mRNA	Yamaguchi et al (1993), Fukao et al (1991, 1995b, 2018)
7 (GK07)	F	Caucasian (USA)	—	—	9 m	Favorable	c.1136G>T (p.Gly379Val) / c.814C>T (p.Gln272* [75% of mRNA], exon 8 skipping [25% of mRNA])	Yamaguchi et al (1993), Fukao et al (1994, 1995b, 2018)
8 (GK08)	F	Chilean (Canada)	+	+[c]	12 m	Favorable	c.2T>A (reduced translation efficiency) homozygous	Daum et al (1973), Fukao et al (1993, 1995b, 2018)
9 (GK08s)	F	Chilean (Canada)	+	+[c]	12 m	Favorable	c.2T>A (reduced translation efficiency) homozygous	Fukao et al (2018)
10 (GK09)	M	Caucasian (Canada)	—	—	17 m	Favorable	c.1163+2T>C (splice donor site; activates cryptic splice site causing c.1163_1164ins GCAG) homozygous	Daum et al (1971, 1973), Fukao et al (1993, 1995b, 2018)
11 (GK10)	M	Vietnamese (Canada)	—	+[c]	6 m	Favorable	c.1006-1G>C (splice acceptor site; causing exon 11 skipping) homozygous	Yamaguchi et al (1993), Fukao et al (1992b, 1995b, 2018)
12 (GK11)	M	Caucasian (Canada)	—	—	25 m	Favorable	c.472A>G (p.Asn158Asp) / c.890C>T (p.Thr297Met)	Wakazono et al (1995), Fukao et al (1995b, 2018)
13 (GK12)	M	Brazilian (Brazil)	—	—	23 m	Impaired	c.99T>A (p.Tyr33*) / (p.Gly405del)	Wajner et al (1992), Yamaguchi et al (1993), Fukao et al (1995b, 2001, 2018)
14 (GK13)	F	Caucasian (Switzerland)	—	—	21 m	Favorable	c.435+1G>A (splice donor site; probably exon 5 skipping) / c.754_755insCT (p.Glu252Alafs*17)	Fukao et al (1995b, 1997, 2018)
15 (GK14)	M	Caucasian (Switzerland)	—	—	21 m	Favorable	c.83_84del (p.Tyr28Cysfs*38) / c.435+1G>A (splice donor site; probably exon 5 skipping)	Fukao et al (1995b, 1997, 2018)
16 (GK15)	M	Caucasian (Canada)	—	—	10 m	Favorable	c.472A>G (p.Asn158Asp) / c.814C>T (p.Gln272* [75% of mRNA], exon 8 skipping [25% of mRNA])	Yamaguchi et al (1993), Fukao et al (1995b, 2018), Sakurai et al (2007)
17 (GK16)	F	Caucasian (USA)	—	—	16 m	Died	c.826+1G>T (splice donor site; causing exon 8 skipping) / c.901G>C (p.Ala301Pro)	Wakazono et al (1995), Fukao et al (1995b, 2018)
18 (GK17)	M	Caucasian (Netherlands)	—	+	5 m	Favorable	c.731-46_752del (splice acceptor site; causing exon 8 skipping) / c.1163+2T>C (splice donor site; activates cryptic splice site causing c.1163_1164ins GCAG))	Fukao et al (1995a&b, 2001, 2018)
19 (GK18)	F	Caucasian (Spain)	—	—	12 m	Favorable	c.371A>G (p.Lys124Arg) homozygous	Fukao et al (2002, 2018)
20 (GK19)	M	Japanese (Japan)	+	+	23 m	Favorable	c.278A>G (Asn93Ser) / c.935T>C (Ile312Thr)	Fukao et al (1996, 1998, 2003a, 2018), Hori et al (2015)
21 (GK19b)	M	Japanese (Japan)	+	+	—	Favorable	c.278A>G (Asn93Ser) / c.935T>C (Ile312Thr)	Fukao et al (1996, 1998, 2003a, 2018)
22 (GK20)	F	Caucasian (Canada)	—	—	22 m	Favorable	c.655T>C (p.Tyr219His) / c.814C>T (p.Gln272* [75% of mRNA], exon 8 skipping [25% of mRNA])	Fukao et al (2018)
23 (GK21)	F	Caucasian (German)	—	—	4 y	Favorable	c.1035_1037del (p.Glu345del) / c.1083dup (p.Ala362Serfs*4)	Sewell et al (1998), Fukao et al (2018)
24 (GK22)	F	Caucasian (Spain)	—	—	7 m	Favorable	c.455G>C (p.Gly152Ala) / c.1061A>T (p.Glu354Val)	Fukao et al (2002, 2018)
25 (GK23)	F	Caucasian (Spain)	—	—	14 m	Favorable	c.433C>G (p.Gln145Glu) homozygous	Riudor et al (1995), Fukao et al (2002, 2018)
26 (GK24)	F	Caucasian (USA)	—	—	3 d	Favorable	c.455G>C (p.Gly152Ala) / c.759T>A (p.Asp253Glu)	Fukao et al (2001, 2018)
27 (GK25)	M	Caucasian (Spain)	—	+	5 m	Favorable	c.380C>T (p.Ala127Val [6% of mRNA], activates cryptic splice acceptor site causing c.336_386 del (p.Leu113_Gly129 del) [94% of mRNA]) homozygous	Merinero et al (1987), Nakamura et al (2001), Fukao et al (2002, 2018)
28 (GK25s)	F	Caucasian (Spain)	—	+	—	Favorable	c.380C>T (p.Ala127Val [6% of mRNA], activates cryptic splice acceptor site causing c.336_386 del	Merinero et al (1987), Fukao et al (2018)

Patient (ID)[a]	Sex	Race (nationality)	Consa-nguin.	Family history	Age at Onset	Neuro-Develop. outcome	Nucleotide (predicted amino acid) changes[b]	References
							(p.Leu113_Gly129 del) [94% of mRNA]) homozygous	
29 (GK27)	M	Sardinian (Italy)	NA	NA	7 m	Favorable	c.844A>C (p.Asn282His) homozygous	Sakurai et al (2007)
30 (GK29)	F	Sardinian (Italy)	NA	NA	14 m	Favorable	c.756_758del (p.Glu252del) homozygous	Sakurai et al (2007)
31 (GK30)	M	Japanese (Japan)	—	—	9 m	Favorable	c.2T>C ([reduced translation efficiency) / c.149del (p.Thr50Asnfs*7)	Fukao et al (2003a&b), Hori et al (2015)
32 (GK31)	M	Japanese (Japan)	NA	NA	18 m	Favorable	c.149del (p.Thr50Asnfs*7) / c.935T>C (Ile312Thr)	Fukao et al (2003a), Hori et al (2015)
33 (GK32)	M	Caucasian (UK)	+	NA	9 m	Impaired	c.218A>C (p.Gln73Pro) / c.623G>A (p.Arg208Gln)	Sakurai et al (2007)
34 (GK33)	M	Caucasian (UK)	+	—	11 m	Favorable	c.377G>C (p.Cys126Ser) homozygous	This paper
35 (GK35)	M	Dutch (Netherlands)	—	—	12 m	Favorable	c.622C>T (p.Arg208*) / c.1163+2T>C (splice donor site; activates cryptic splice site causing c.1163_1164ins GCAG)	Fukao et al (2010a), Grünert et al (2017)
36 (GK36)	F	Dutch (Netherlands)	—	+	—	Impaired	c.622C>T (p.Arg208*) / c.1006-2A>C (splice acceptor site; causing exon 11 skipping)	Fukao et al (2010a), Grünert et al (2017)
37 (GK36b)	M	Dutch (Netherlands)	—	+	—	Favorable	c.622C>T (p.Arg208*) / c.1006-2A>C (splice acceptor site; causing exon 11 skipping)	Grünert et al (2017)
38 (GK37)	M	Arab (Israel)	+	+[c]	13 m	Favorable	c.1241_1245delinsGT (p.Asn414_Gly415delinsSer) homozygous	Gibson et al (1996)
39 (GK38)	M	French (Canada)	NA	NA	2 y	Favorable	c.655T>C (p.Tyr219His) / c.814C>T (p.Gln272* [75% of mRNA], exon 8 skipping [25% of mRNA])	Sakurai et al (2007)
40 (GK39)		East Indian (Canada)	—	—	11 m	Favorable	NA	Gibson et al (1997)
41 (GK40)	F	African (USA)	NA	NA	6 m	Favorable	c.473A>G (p.Asn158Ser) / c.1059T>A (p.Asn353Lys)	Sakurai et al (2007)
42 (GK41)	M	(USA)	NA	NA	14 m	Favorable	c.(72+1_73-1)_(344+1_345-1)del (del exons 2-4 [≈ 10% of mRNA]), c.(72+1_73-1)_(435+1_436-1)del (del exons 2-5 [≈ 90% of mRNA])	Zhang et al (2006)
43 (GK43)	F	Lebanese (Australia)	+	—	2.7 y	Favorable	c.1124A>G (activates a cryptic splice donor site causing c.1120_1163del [89% of mRNA], p.Asn375Ser [11% of mRNA]) homozygous	Fukao et al (2008)
44 (GK44)	F	Caucasian (Australia)	—	—	9 m	Favorable	c.(120+1_121-1)_(344+1_345-1)del (del exons 3-4) / c.602C>T (p.Ala201Val)	Fukao et al (2013)
45 (GK45)	M	Armenian (Lebanon)	+	—	15 m	Favorable	c.395C>G (p.Ala132Gly) homozygous	Zhang et al (2004)
46 (GK46)	M	Vietnamese (France)	—	—	18 m	Favorable	c.52dup (p.Leu18Profs*49) / c.455G>C (p.Gly152Ala)	Zhang et al (2004), Paquay et al (2017)
47 (GK47)	M	Caucasian (France)	—	—	19 m	Favorable	c.455G>C (p.Gly152Ala) / c.1016_1018dup (p.Asp339dup)	Renom et al (2000), Zhang et al (2004), Paquay et al (2017)
48 (GK48)	F	Caucasian (France)	+	—	10 m	Favorable	c.(730+1_731-1)_(940+1_941-1)dup (tandem duplication of exons 8-9)	Fukao et al (2007)
49 (GK49)	M	(France)	+	—	15 m	Favorable	c.1189C>G (p.His397Asp) homozygous	Zhang et al (2004), Paquay et al (2017)
50 (GK50)	M	(France)	—	—	2 d	Favorable	c.826+1G>T (splice donor site; causing exon 8 skipping) homozygous	Zhang et al (2004), Paquay et al (2017)
51 (GK51)	F	(France)	NA	NA	11 m	Favorable	c.760G>A (p.Glu254Lys) / ?	Paquay et al (2017)
52 (GK59)	M	German/ Bosnian	—	+	2 y	Favorable	c.826+1G>T (splice donor site; causing exon 8 skipping) / c.949G>A (affects ESE sequence causing exon 10 skipping [80% of mRNA], Asp317Asn [20% of mRNA])	Grünert et al (2017)
53 (GK63)	F	German (Germany)	—	—	11 m	Favorable	c.472A>G (p.Asn158Asp) / c.949G>A (affects ESE sequence causing exon 10 skipping [80% of mRNA], Asp317Asn [20% of mRNA])	Otsuka et al (2016), Grünert et al (2017)
54 (GK64)	F	Japanese (Japan)	—	—	7 m	Favorable	c.556G>T (p.Asp186Tyr) / c.951C>T (affects ESE sequence causing exon 10 skipping [≈ 40% of mRNA], p.317Asp= [≈ 60% of mRNA])	Fukao et al (2010b), Hori et al (2015)
55 (GK65)	F	French (France)	—	+	2 y	Favorable	c.578T>C (p.Met193Thr) / c.826+5G>T (splice donor site; causing exon 8 skipping)	Thummler et al (2010), Paquay et al (2017)
56 (GK65b)	M	French (France)	—	+	6 y	Favorable	c.578T>C (p.Met193Thr) / c.826+5G>T (splice donor site; causing exon 8 skipping)	Thummler et al (2010), Paquay et al (2017)

Patient (ID)[a]	Sex	Race (nationality)	Consa-nguin.	Family history	Age at Onset	Neuro-Develop. outcome	Nucleotide (predicted amino acid) changes[b]	References
57 (GK66)	F	Dutch	—	+	3.7 y	Favorable	c.462_482delinsTCCTC (p.Glu154Aspfs*4) / c.547G>A (p.Gly183Arg)	Grünert et al (2017)
58 (GK66s)	F	Dutch	—	+	—	Favorable	c.462_482delinsTCCTC (p.Glu154Aspfs*4) / c.547G>A (p.Gly183Arg)	Grünert et al (2017)
59 (GK69)	F	Japanese (Japan)	NA	NA	9 m	Favorable	c.431A>C (p.His144Pro) / c.1168T>C (p.Ser390Pro)	Fukao et al (2012), Hori et al (2015)
60 (GK70)	M	Vietnamese (Vietnam)	—	—	3 y	Died (5.5 y of age)	c.163_167delinsAA (p.Phe55_Leu56delinsLys) / c.622C>T (p.Arg208*)	Fukao et al (2010a), Nguyen et al (2017)
61 (GK72)	M	Vietnamese (Vietnam)	—	+[c]	12 m	Favorable	c.622C>T (p.Arg208*) homozygous	Fukao et al (2010a), Nguyen et al (2017)
62 (GK73)	M	Vietnamese (Vietnam)	—	+[c]	13 m	Favorable	c.622C>T (p.Arg208*) homozygous	Fukao et al (2010a), Nguyen et al (2017)
63 (GK74)	M	Vietnamese (Vietnam)	—	—	18 m	Died (2 y of age)	c.622C>T (p.Arg208*) / c.1006-1G>C (splice acceptor site; causing exon 11 skipping)	Fukao et al (2010a), Nguyen et al (2017)
64 (GK75)	M	Vietnamese (Vietnam)	—	+[c]	11 m	Favorable	c.622C>T (p.Arg208*) homozygous	Fukao et al (2010a), Nguyen et al (2017)
65 (GK76)	M	Vietnamese (Vietnam)	—	—	13 m	Favorable	c.622C>T (p.Arg208*) homozygous	Fukao et al (2010a), Nguyen et al (2017)
66 (GK77)		Japanese (Japan)	—	+	3 y	Favorable	c.431A>C (p.His144Pro) homozygous	Fukao et al (2012), Hori et al (2015)
67 (GK77b)	M	Japanese (Japan)	—	+	3 y	Died	c.431A>C (p.His144Pro) homozygous	Fukao et al (2012), Hori et al (2015)
68 (GK79)	F	Vietnamese (Vietnam)	—	—	12 m	Favorable	c.622C>T (p.Arg208*) homozygous	Fukao et al (2010a), Nguyen et al (2017)
69 (GK80)	F	Vietnamese (Vietnam)	—	—	9 m	Favorable	c.622C>T (p.Arg208*) homozygous	Fukao et al (2010a), Nguyen et al (2017)
70 (GK84)	M	French-Canadian (Canada)	—	—	5 y[e]	Impaired	c.455G>C (p.Gly152Ala) / c.472A>G (p.Asn158Asp)	Buhas et al (2013)
71 (GK86)	F	Vietnamese (Vietnam)	—	—	10 m	Favorable	c.1006-1G>C (splice acceptor site; causing exon 11 skipping) homozygous	Nguyen et al (2017)
72 (GK87)	F	Vietnamese (Vietnam)	—	—	11 m	Favorable	c.622C>T (p.Arg208*) homozygous	Nguyen et al (2017)
73 (GK89)	F	Vietnamese (Vietnam)	—	—	12 m	Favorable	c.1006-1G>C (splice acceptor site; causing exon 11 skipping) homozygous	Nguyen et al (2017)
74 (GK90)	M	Vietnamese (Vietnam)	—	—	7 m	Favorable	c.622C>T (p.Arg208*) / c.1229C>T (p.Ala410Val)	Nguyen et al (2017)
75 (GK91)	M	Vietnamese (Vietnam)	—	—	9 m	Favorable	c.622C>T (p.Arg208*) homozygous	Nguyen et al (2017)
76 (GK93)	M	Vietnamese (Vietnam)	—	+	17 m	Favorable	c.622C>T (p.Arg208*) homozygous	Nguyen et al (2017)
77 (GK93b)	M	Vietnamese (Vietnam)	—	+	22 m	Favorable	c.622C>T (p.Arg208*) homozygous	Nguyen et al (2017)
78 (GK94)	M	Indian (India)	+	—	10 m	Favorable	c.254_256del (p.Glu85del) homozygous	Abdelkreem et al (2017a)
79 (GK95)	M	Indian (India)	+	—	11 m	Impaired	c.578T>G (p.Met193Arg) homozygous	Akella et al (2014)
80 (GK98)	M	Indian (India)	—	—	4 m	Impaired	c.578T>G (p.Met193Arg) homozygous	Abdelkreem et al (2017a)
81 (GK99)	M	Indian (India)	—	—	14 m	Favorable	c.578T>G (p.Met193Arg) homozygous	Abdelkreem et al (2017a)
82 (GK100)	F	Vietnamese (Vietnam)	—	—	9 m	Favorable	c.1006-1G>C (splice acceptor site; causing exon 11 skipping) / g.20623_29833delinsGTAA (probably del exons 6-11)	Nguyen et al (2017)
83 (GK101)	M	Vietnamese (Vietnam)	—	+	14 m	Favorable	c.622C>T (p.Arg208*) homozygous	Nguyen et al (2017)
84 (GK102)	M	Vietnamese (Vietnam)	—	—	20 m	Favorable	c.622C>T (p.Arg208*) / c.851G>A (p.Ser284Asn)	Nguyen et al (2017)
85 (GK103)	M	Vietnamese (Vietnam)	—	—	20 m	Favorable	c.622C>T (p.Arg208*) / c.1006-1G>C (splice acceptor site; causing exon 11 skipping)	Nguyen et al (2017)
86 (GK104)	F	Vietnamese (Vietnam)	—	+	16 m	Favorable	c.622C>T (p.Arg208*) / c.1032dup (p.Glu345Argfs*10)	Nguyen et al (2017)
87 (GK104b)	M	Vietnamese (Vietnam)	—	+	—	Favorable	c.622C>T (p.Arg208*) / c.1032dup (p.Glu345Argfs*10)	Nguyen et al (2017)
88 (GK105)	M	Vietnamese (Vietnam)	—	—	11 m	Favorable	c.622C>T (p.Arg208*) / c.1006-1G>C (splice acceptor site; causing exon 11 skipping)	Nguyen et al (2017)
89 (GK106)	F	Persian (Iran)	+	—	1 m	Favorable	c.1035_1037del (p.Glu345del) homozygous	Alijanpour et al (2019)
90 (GK108)	F	Indian (India)	+	—	11 m	Died (11 m of age)	c.578T>G (p.Met193Arg) homozygous	Abdelkreem et al (2017a)

Patient (ID)[a]	Sex	Race (nationality)	Consa-nguin.	Family history	Age at Onset	Neuro-Develop. outcome	Nucleotide (predicted amino acid) changes[b]	References
91 (GK109)	M	Indian (India)	—	—	9 m	Impaired	c.1013_1016dup (p.Asp339Glufs*17) homozygous	Abdelkreem et al (2017a)
92 (GK110)	F	Indian (India)	+	—	11 m	Favorable	c.730+1G>A (splice donor site; probably exon 7 skipping) homozygous	Abdelkreem et al (2017a)
93 (GK111)	M	Indian (India)	—	—	6 m	Favorable	c.578T>G (p.Met193Arg) / c.968T>C (p.Ile323Thr)	Abdelkreem et al (2017a)
94 (GK112)	F	Indian (India)	+	—	7 m	Favorable	c.1124A>G (activates a cryptic splice donor site causing c.1120_1163del [89% of mRNA], p.Asn375Ser [11% of mRNA]) homozygous	Abdelkreem et al (2017a)
95 (GK113)	M	Indian (India)	+	—	12 m	Impaired	c.643_644delinsAA (p.Ala215Asn) homozygous	Abdelkreem et al (2017a)
96 (GK114)	M	Indian (India)	—	—	19 m	Favorable	c.578T>G (p.Met193Arg) homozygous	Abdelkreem et al (2017a)
97 (GK115)	F	Vietnamese (Vietnam)	—	—	14 m	Favorable	c.622C>T (p.Arg208*) / c.1006-1G>C (splice acceptor site; causing exon 11 skipping)	Nguyen et al (2017)
98 (GK116)	M	Vietnamese (Vietnam)	—	—	14 m	Favorable	c.622C>T (p.Arg208*) / c.1006-1G>C (splice acceptor site; causing exon 11 skipping)	Nguyen et al (2017)
99 (GK117)	F	Vietnamese (Vietnam)	—	—	8 m	Died (8 m of age)	c.622C>T (p.Arg208*) homozygous	Nguyen et al (2017)
100 (GK118)	F	Vietnamese (Vietnam)	—	+	3 y	Favorable	c.1A>G (reduced translation efficiency) homozygous	Nguyen et al (2017)
101 (GK118s)	F	Vietnamese (Vietnam)	—	+	12 m	Favorable	c.1A>G (reduced translation efficiency) homozygous	Nguyen et al (2017)
102 (GK119)	M	Vietnamese (Vietnam)	—	—	23 m	Favorable	c.1006-1G>C (splice acceptor site; causing exon 11 skipping) homozygous	Nguyen et al (2017)
103 (GK120)	F	Vietnamese (Vietnam)	—	—	14 m	Favorable	c.622C>T (p.Arg208*) homozygous	Nguyen et al (2017)
104 (GK121)	F	Vietnamese (Vietnam)	—	—	6 m	Favorable	c.622C>T (p.Arg208*) homozygous	Nguyen et al (2017)
105 (GK122)	M	Arab (Libya)	+	+[c]	4 y	Favorable	c.674C>A (Ala225Glu) homozygous	Abdelkreem et al (2017b)
106 (GK124)	F	Hispanic (USA)	NA	—	5 y	Impaired	c.941-9T>A (splice acceptor site (causing exon 10 skipping in 90% of transcripts) homozygous	Sasai et al (2017)
107 (GK126)	F	Indian (India)	+	—	10 m	Favorable	c.121-13T>A (splice acceptor site; causing exon 3 skipping in >90% of mRNA) homozygous	Aoyama et al (2017)
108	F	Arab (KSA)	+	+	7 m[e]	Favorable	NA	Ozand et al (1994)
109	F	Arab (KSA)	+	+	6 m[e]	Impaired	NA	Ozand et al (1994)
110	M	Arab (KSA)	+	—	8 m[e]	Impaired	NA	Ozand et al (1994)
111	F	Indian (Canada)	—	—	11 m	Favorable	NA	Gibson and Feigenbaum (1997)
112	M	Arab (Tunisia)	+	+[c]	10 m	NA	NA	Monastiri et al (1999)
113	M	(Turkey)	+	—	7 y	Impaired	NA	Yalçinkaya et al (2001)
114	M	(Slovakia)	NA	NA	21 m	Favorable	c.578T>C (p.Met193Thr) / c.1040T>C (p.Ile347Thr)	Mrazova et al (2005)
115	F	(Italy)	+	+	—	NA	c.1189C>G (p.His397Asp) homozygous	Catanzono et al (2010)
116 (115s)	F	(Italy)	+	+	18 m	NA	c.1189C>G (p.His397Asp) homozygous	Catanzono et al (2010)
117	M	Hispanic (USA)	—	+	10 m	Favorable	c.52dup (p.Leu18Profs*49) / c.473A>G (p.Asn158Ser)	Sarafoglou et al (2011)
118 (117s)	F	Hispanic (USA)	—	+	—	Favorable	c.52dup (p.Leu18Profs*49) / c.473A>G (p.Asn158Ser)	Sarafoglou et al (2011)
119	M	White/Hispanic (USA)	NA	NA	—	Favorable	c.286C>T (p.Gln96*) / c.622C>T (p.Arg208*)	Sarafoglou et al (2011)
120	NA	Indian (UAE)	NA	NA	NA	NA	c.460G>A (p.Glu154Lys) / c.578T>G (p.Met193Arg)	Ali et al (2011)
121	NA	Arab (UAE)	NA	NA	NA	NA	c.86_87dup (p.Glu30Trpfs*11) / c.854C>T (p.Thr285Ile)	Al-Shamsi et al (2014), Al-Jasmi et al (2016)
122	M	(China)	—	NA	12 m	Favorable	c.354_355delinsG (p.Cys119Valfs*4) / c.1006-1G>A (splice acceptor site; probably exon 11 skipping)	Law et al (2015)
123	M	Chinese (China)	—	+	NA	NA	c.622C>T (p.Arg208*) / c.653C>T (p.Ser218Phe)	Wen et al (2016)
124	M	(Turkey)	+	+	8 m	Impaired	c.949G>A (affects ESE sequence causing exon 10 skipping [80% of mRNA], Asp317Asn [20% of mRNA]) homozygous	Köse et al (2016), Grünert et al (2017)

Patient (ID)[a]	Sex	Race (nationality)	Consa-nguin.	Family history	Age at Onset	Neuro-Develop. outcome	Nucleotide (predicted amino acid) changes[b]	References
125 (124s)	F	(Turkey	+	+	—	Favorable	c.949G>A (affects ESE sequence causing exon 10 skipping [80% of mRNA], Asp317Asn [20% of mRNA]) homozygous	Köse et al (2016), Grünert et al (2017)
126	NA	(France)	NA	NA	10 m	Favorable	NA	Paquay et al (2017)
127	NA	(France)	—	NA	12 m[e]	Impaired	c.79A>T (p.Arg27*) / c.1223_1226dup (p.Ala410Serfs*51)	Paquay et al (2017)
128	NA	(France)	NA	NA	15 m	Favorable	c.446del (p.Val149Glyfs*14) / c.1163G>A (splice donor site with probable exon 11 skipping)	Paquay et al (2017)
129	NA	(France)	NA	NA	2.5 y	Favorable	NA	Paquay et al (2017)
130	NA	(France)	NA	NA	18 m	Favorable	c.83_84del (p.Tyr28Cysfs*38) homozygous	Paquay et al (2017)
131	NA	(France)	NA	NA	2 y	Favorable	c.1033_1034del (p.Glu345Argfs*9) homozygous	Paquay et al (2017)
132	NA	(France)	NA	NA	10 m	Favorable	c.1033_1034del (p.Glu345Argfs*9) homozygous	Paquay et al (2017)
133	NA	(France)	NA	NA	19 m	Favorable	c.534G>T (p.Leu178Phe) / c.1059T>G (p.Asn353Lys)	Paquay et al (2017)
134	NA	(France)	NA	NA	2 y	Impaired	c.760G>A (p.Glu254Lys) homozygous	Paquay et al (2017)
135	NA	(France)	NA	NA	22 m	Favorable	c.414_415del (p.Leu140Tyrfs*36) homozygous	Paquay et al (2017)
136	NA	(France)	—	NA	11 m	Favorable	c.83_84del (p.Tyr28Cysfs*38) homozygous	Paquay et al (2017)
137	NA	(France)	NA	+	11 m	Impaired	c.947_949dup (p.Ala316dup) / c.1167G>A (p.Met389Ile)	Paquay et al (2017)
138 (137s)	NA	(France)	NA	+	—	Favorable	c.947_949dup (p.Ala316dup) / c.1167G>A (p.Met389Ile)	Paquay et al (2017)
139	NA	(France)	NA	NA	22 m	Favorable	c.765A>T (p.Glu255Asp) / c.814C>T (p.Gln272* [75% of mRNA], exon 8 skipping [25% of mRNA])	Fukao et al (1994), Paquay et al (2017)
140	M	Tamil (Sri Lanka)	+	NA	—	Favorable	c.578T>G (p.Met193Arg) homozygous	Grünert et al (2017)
141	F	Turkish	+	NA	2.25 y	Favorable	c.1040T>C (p.Ile347Thr) homozygous	Grünert et al (2017)
142	F	Turkish	—	NA	5 m	Favorable	c.940+1G>T (splice donor site; probably exon 9 skipping) homozygous	Grünert et al (2017)
143	M	Turkish	+	NA	11 m	Favorable	NA	Grünert et al (2017)
144	F	Afghan	+	NA	—	Impaired	c.370A>G (p.Lys124Glu) homozygous	Grünert et al (2017)
145	F	German	—	NA	18 m	Favorable	c.301C>A (p.Gln101Lys) / c.1253G>A (p.Gly418Asp)	Grünert et al (2017)
146	M	Turkish	+	NA	—	Favorable	c.1163+2T>C (splice donor site; activates cryptic splice site causing c.1163_1164ins GCAG) homozygous	Grünert et al (2017)
147	F	Turkish	+	NA	—	Favorable	c.826+5_826+9del (splice donor site (probably exon 8 skipping) homozygous	Grünert et al (2017)
148	M	Turkish	+	NA	9 m	Favorable	c.334+1G>A (splice donor site; probably exon 4 skipping) homozygous	Grünert et al (2017)
149	F	Turkish	—	NA	12 m	Favorable	c.622C>T (p.Arg208*) homozygous	Grünert et al (2017)
150	M	Turkish	—	+	—	Favorable	c.622C>T (p.Arg208*) homozygous	Grünert et al (2017)
151	M	Chinese (China)	NA	NA	8 m	Favorable	c.829A>C (p.Thr277Pro) / c.997G>C (p.Ala333Pro)	Su et al (2017)
152	F	Chinese (China)	NA	NA	8 m	Favorable	c.890C>A (p.Thr297Lys) / c.121-3C>G (splice acceptor site; probably exon 3 skipping)	Su et al (2017)
153	F	Chinese (China)	NA	NA	13 m	Favorable	c.83_84del (p.Tyr28Cysfs*65) / c.1006-1G>C (splice acceptor site; causing exon 11 skipping)	Su et al (2017)
154	M	Chinese (China)	NA	NA	9 m	Favorable	c.1006-1G>C (splice acceptor site; causing exon 11 skipping) homozygous	Su et al (2017)
155	F	(USA)	NA	NA	28 m	Favorable	c.1006-1G>C (splice acceptor site; causing exon 11 skipping) / c.1160T>C (p.Ile387Thr)	Wojcik et al (2018)
156	M	(USA)	NA	NA	6 m	Impaired	c.299G>A (p.Gly100Glu) / c.1006-2A>C (splice acceptor site; causing exon 11 skipping)	Wojcik et al (2018)
157	F	Chinese (China)	NA	—	8 m	Favorable	c.547G>A (p.Gly183Arg) homozygous	Hu et al (2017)
158	F	Persian (Iran)	+	+	2 m	Impaired	c.664A>C (p.Ser222Arg) homozygous	Vakili and Hashemian (2018)

Patient (ID)[a]	Sex	Race (nationality)	Consa-nguin.	Family history	Age at Onset	Neuro-Develop. outcome	Nucleotide (predicted amino acid) changes[b]	References
159	M	Indian (India)	+	+	9 m	Impaired	c.764A>C (p.Glu255Ala) homozygous	Sundaram et al (2018)

[a] GK number refers to an internal identifier for patients with mitochondrial acetoacetyl-CoA thiolase deficiency whose *ACAT1* variants were identified at Gifu University (Gifu, Japan). Sib pairs are denoted by b (brother) and s (sister) following patient codes.
[b] Numbering of nucleotide and predicted amino acid changes follows the HGVS nomenclature (version 15.11; http://varnomen.hgvs.org; den Dunnen et al., 2016) using *ACAT1* NCBI reference sequences (**NM_000019.3**, **NG_009888.1**, and **NP_000010.1**) with +1 as the number of the A of the ATG initiation codon. No sequence information is available for 10 cases.
[c] History of death of sibling/s
[d] GK05 is the father of GK04
[e] Neurological impairment has preceded the ketoacidotic episode.
Abbreviations: d, day; F, female; KSA, Kingdom Saudi Arabia; M, male; m, month; NA, not applicable; UAE, Arab United Emirates; USA, United states of America; y, year.

References

Abdelkreem E, Akella RR, Dave U, Sane S, Otsuka H, Sasai H, Aoyama Y, Nakama M, Ohnishi H, Mahmoud S, Abd El Aal M, Fukao T (2017a) Clinical and Mutational Characterizations of Ten Indian patients with Beta-ketothiolase Deficiency. *JIMD Rep* 35:59–65. doi: 10.1007/8904_2016_26.

Abdelkreem E, Alobaidy H, Aoyama Y, Mahmoud S, Abd El Aal M, Fukao T (2017b) Two Libyan siblings with beta-ketothiolase deficiency: A case report and review of literature. *Egypt J Med Hum Genet* 18(2): 199–203. doi: 10.1016/j.ejmhg.2016.11.001.

Akella RR, Aoyama Y, Mori C, Lingappa L, Cariappa R, Fukao T (2014) Metabolic encephalopathy in beta-ketothiolase deficiency: the first report from India. *Brain Dev* 36(6): 537–540. doi: 10.1016/j.braindev.2013.07.007.

Alijanpour M, Sasai H, Abdelkreem E, Ago Y, Soleimani S, Moslemi L, Yamaguchi S, Rezapour M, Hakimi MT, Matsumoto H, Fukao T (2019) Beta-Ketothiolase deficiency: A case with unusual presentation of nonketotic hypoglycemic episodes due to coexistent probable secondary carnitine deficiency. *JIMD Rep* 46(1): 23–27. doi: 10.1002/jmd2.12022.

Al-Jasmi FA, Al-Shamsi A, Hertecant JL, Al-Hamad SM, Souid AK (2016) Inborn Errors of Metabolism in the United Arab Emirates: Disorders Detected by Newborn Screening (2011-2014). *JIMD Rep* 28: 127–135. doi: 10.1007/8904_2015_512.

Ali BR, Hertecant JL, Al-Jasmi FA, Hamdan MA, Khuri SF, Akawi NA, Al-Gazali LI (2011) New and known mutations associated with inborn errors of metabolism in a heterogeneous Middle Eastern population. *Saudi Med J* 32(4): 353-359. PMID: 21483992.

Al-Shamsi A, Hertecant JL, Al-Hamad S, Souid AK, Al-Jasmi F (2014) Mutation Spectrum and Birth Prevalence of Inborn Errors of Metabolism among Emiratis: A study from Tawam Hospital Metabolic Center, United Arab Emirates. *Sultan Qaboos Univ Med J* 14(1): e42–49. PMID: 24516753.

Aoyama A, Sasai1H, Abdelkreem E, Otsuka H, Nakama M, Kumar S, Aroor S, Shukla A, Fukao T (2017) A novel mutation (c.121-13T>A) in the polypyrimidine tract of the splice acceptor site of intron 2 causes exon 3 skipping in mitochondrial acetoacetyl-CoA thiolase gene. *Mol Med Rep* 15(6):3879-3884. doi: 10.3892/mmr.2017.6434.

Buhaş D, Bernard G, Fukao T, Décarie JC, Chouinard S, Mitchell GA (2013) A treatable new cause of chorea: beta-ketothiolase deficiency. *Mov Disord* 28(8): 1054–1056. doi: 10.1002/mds.25538.

Catanzano F, Ombrone D, Di Stefano C, Rossi A, Nosari N, Scolamiero E, Tandurella I, Frisso G, Parenti G, Ruoppolo M, Andria G, Salvatore F (2010) The first case of mitochondrial acetoacetyl-CoA thiolase deficiency identified by expanded newborn metabolic screening in Italy: the importance of an integrated diagnostic approach. *J Inherit Metab Dis* 33(Suppl 3): S91–94. doi: 10.1007/s10545-009-9028-3.

Daum RS, Lamm PH, Mamer OA, Scriver CR (1971) A ‘‘new’’ disorder of isoleucine catabolism. *Lancet* 2(7737): 1289–1290. doi: 10.1016/S0140-6736(71)90605-2.

Daum RS, Scriver CR, Mamer OA, Delvin E, Lamm P, Goldman H (1973) An inherited disorder of isoleucine catabolism causing accumulation of alpha-methylacetoacetate and alpha-methyl-beta-hydroxybutyrate, and intermittent metabolic acidosis. *Pediatr Res* 7: 149–160. doi: 10.1203/00006450-197303000-00007.

den Dunnen JT, Dalgleish R, Maglott DR, Hart RK, Greenblatt MS, McGowan-Jordan J, Roux AF, Smith T, Antonarakis SE, Taschner PE (2016) HGVS Recommendations for the Description of Sequence Variants: 2016 Update. *Hum Mutat*. 37(6):564-569. doi: 10.1002/humu.22981.

Fukao T, Yamaguchi S, Kano M, Orii T, Fujiki Y, Osumi T, Hashimoto T (1990) Molecular cloning and sequence of the complementary DNA encoding human mitochondrial acetoacetyl-coenzyme A thiolase and study of the variant enzymes in cultured fibroblasts from patients with 3-ketothiolase deficiency. *J Clin Invest* 86(6):2086-2092. doi: 10.1172/JCI114946.

Fukao T, Yamaguchi S, Tomatsu S, Orii T, Fraudienst-Egger G, Schrod L, Osumi T, Hashimoto T (1991) Evidence for structural mutation (^{347}Ala to Thr) in a German family with 3-ketothiolase deficiency. *Biochem Biophys Res Commun* 179(1): 124–129. doi: 10.1016/0006-291X(91)91343-B.

Fukao T, Yamaguchi S, Orii T, Schutgens RBH, Osumi T, Hashimoto T (1992a) Identification of three mutant alleles of the gene for mitochondrial acetoacetyl-CoA thiolase: A complete analysis of two generations of a family with 3-ketothiolase deficiency. *J Clin Invest* 89(2): 474–479. doi: 10.1172/JCI115608.

Fukao T, Yamaguchi S, Orii T, Osumi T, Hashimoto T (1992b) Molecular basis of 3-ketothiolase deficiency: identification of an AG to AC substitution at the splice acceptor site of intron 10 causing exon 11 skipping. *Biochim Biophys Acta* 1139(3): 184–188. doi: 10.1016/0925-4439(92)90132-7.

Fukao T, Yamaguchi S, Scriver CR, Dunbar G, Wakazono A, Kano M, Orii T, Hashimoto T (1993) Molecular studies of mitochondrial acetoacetyl-coenzyme A thiolase in two original families. *Hum Mutat* 2(3): 214–220. doi: 10.1002/humu.1380020310.

Fukao T, Yamaguchi S, Wakazono A, Orii T, Hoganson G, Hashimoto T (1994) Identification of a novel exonic mutation at -13 from 5‘ splice site causing exon skipping in a girl with mitochondrial acetoacetyl-coenzyme A thiolase deficiency. *J Clin Invest* 93(3): 1035–1041. doi: 10.1172/JCI117052.

Fukao T, Song X-Q, Yamaguchi S, Orii T, Wanders RJA, Poll-The BT, Hashimoto T (1995a) Mitochondria1 acetoacetyl-coenzyme A thiolase gene: A novel 68-bp deletion involving 3’ splice site of intron 7, causing exon 8 skipping in a Caucasian patient with beta-ketothiolase deficiency. *Hum Mutat* 5(1): 94–96. doi: 10.1002/humu.1380050113.

Fukao T, Yamaguchi S, Orii T, Hashimoto T (1995b) Molecular basis of β-ketothiolase deficiency: Mutations and polymorphisms in the human mitochondrial acetoacetyl-coenzyme a thiolase gene. *Hum Mutat* 5(2): 113–120. doi: 10.1002/humu.1380050203.

Fukao T, Kodama A, Aoyanagi N, Tsukino R, Uemura S, Song XQ, Watanebe H, Kuhara T, Matsumoto I, Orii T, Kondo N (1996) Mild form of beta-ketothiolase deficiency (mitochondrial acetoacetyl-CoA thiolase deficiency) in two Japanese siblings: identification of detectable residual activity and cross-reactive material in EB-transformed lymphocytes. *Clin Genet* 50(4): 263–266. doi: 10.1111/j.1399-0004.1996.tb02641.x.

Fukao T, Xiang-Qian S, Yamaguchi S, Kondo N, Orii T, Matthieu JM, Bachmann C, Hashimoto T (1997) Identification of three novel frameshift mutations (83delAT, 754insCT, and 435 + 1G to A) of mitochondrial acetoacetyl-coenzyme A thiolase gene in two Swiss patients with CRM-negative beta-ketothiolase deficiency. *Hum Mutat* 9(3): 277–279. doi: 10.1002/(SICI)1098-1004(1997)9:33.0.CO;2-#.

Fukao T, Nakamura H, Song XQ, Nakamura K, Orii KE, Kohno Y, Kano M, Yamaguchi S, Hashimoto T, Orii T, Kondo N (1998) Characterization of N93S, I312T, and A333P missense mutations in two Japanese families with mitochondrial acetoacetyl-CoA thiolase deficiency. *Hum Mutat* 12(4): 245–254. doi: 10.1002/(SICI)1098-1004(1998)12:4<245::AID-HUMU5>3.0.CO;2-E.

Fukao T, Scriver CR, Kondo N; T2 Collaborative Working Group (2001) The clinical phenotype and outcome of mitochondrial acetoacetyl-CoA thiolase deficiency (beta-ketothiolase or T2 deficiency) in 26 enzymatically proved and mutation-defined patients. *Mol Genet Metab* 72(2): 109–114. doi: 10.1006/mgme.2000.3113.

Fukao T, Nakamura H, Nakamura K, Perez-Cerda C, Baldellou A, Barrionuevo CR, Castello FG, Kohno Y, Ugarte M, Kondo N (2002) Characterization of six mutations in five Spanish patients with mitochondrial acetoacetyl-CoA thiolase deficiency: effects of amino acid substitutions on tertiary structure. *Mol Genet Metab* 75(3): 235-243. doi: 10.1006/mgme.2001.3288.

Fukao T, Zhang GX, Sakura N, Kubo T, Yamaga H, Hazama A, Kohno Y, Matsuo N, Kondo M, Yamaguchi S, Shigematsu Y, Kondo N (2003a) The mitochondrial acetoacetyl-CoA thiolase (T2) deficiency in Japanese patients: urinary organic acid and blood acylcarnitine profiles under stable conditions have subtle abnormalities in T2-deficient patients with some residual T2 activity. *J Inherit Metab Dis* 26(5): 423–431. doi: 10.1023/A:1025117226051.

Fukao T, Matsuo N, Zhang GX, Urasawa R, Kubo T, Kohno Y, Kondo N (2003b) Single base substitutions at the initiator codon in the mitochondrial acetoacetyl-CoA thiolase (ACAT1/T2) gene result in production of varying amounts of wild-type T2 polypeptide. *Hum Mutat* 21(6): 587–592. doi: 10.1002/humu.10209.

Fukao T, Zhang G, Rolland MO, Zabot MT, Guffon N, Aoki Y, Kondo N (2007) Identification of an Alu-mediated tandem duplication of exons 8 and 9 in a patient with mitochondrial acetoacetyl-CoA thiolase (T2) deficiency. *Mol Genet Metab* 92(4): 375–378. doi: 10.1016/j.ymgme.2007.07.007.

Fukao T, Boneh A, Aoki Y, Kondo N (2008) A novel single-base substitution (c.1124A>G) that activates a 5-base upstream cryptic splice donor site within exon 11 in the human mitochondrial acetoacetyl-CoA thiolase gene. *Mol Genet Metab* 94(4): 417–421. doi: 10.1016/j.ymgme.2008.04.014.

Fukao T, Nguyen HT, Nguyen NT, Vu DC, Can NT, Pham AT, Nguyen KN, Kobayashi H, Hasegawa Y, Bui TP, Niezen-Koning KE, Wanders RJ, de Koning T, Nguyen LT, Yamaguchi S, Kondo N (2010a) A common mutation, R208X, identified in Vietnamese patients with mitochondrial acetoacetyl-CoA thiolase (T2) deficiency. *Mol Genet Metab* 100(1): 37–41. doi: 10.1016/j.ymgme.2010.01.007.

Fukao T, Horikawa R, Naiki Y, Tanaka T, Takayanagi M, Yamaguchi S, Kondo N (2010b) A novel mutation (c.951C>T) in an exonic splicing enhancer results in exon 10 skipping in the human mitochondrial acetoacetyl-CoA thiolase gene. *Mol Genet Metab* 100(4): 339–344. doi: 10.1016/j.ymgme.2010.03.012.

Fukao T, Maruyama S, Ohura T, Hasegawa Y, Toyoshima M, Haapalainen AM, Kuwada N, Imamura M, Yuasa I, Wierenga RK, Yamaguchi S, Kondo N (2012) Three Japanese Patients with Beta-Ketothiolase Deficiency Who Share a Mutation, c.431A>C (H144P) in ACAT1 : Subtle Abnormality in Urinary Organic Acid Analysis and Blood Acylcarnitine Analysis Using Tandem Mass Spectrometry. *JIMD Rep* 3: 107–115. doi: 10.1007/8904_2011_72.

Fukao T, Aoyama Y, Murase K, Hori T, Harijan RK, Wierenga RK, Boneh A, Kondo N (2013) Development of MLPA for human ACAT1 gene and identification of a heterozygous Alu-mediated deletion of exons 3 and 4 in a patient with mitochondrial acetoacetyl-CoA thiolase (T2) deficiency. *Mol Genet Metab* 110(1-2): 184–187. doi: 10.1016/j.ymgme.2013.07.004.

Fukao T, Sasai H, Aoyama Y, Otsuka H, Ago Y, Matsumoto H, Abdelkreem E (2018) Recent advances in understanding beta-ketothiolase (mitochondrial acetoacetyl-CoA thiolase, T2) deficiency. *J Hum Genet* 64(2): 99-111. doi: 10.1038/s10038-018-0524-x.

Gibson KM, Elpeleg ON, Bennett MJ (1996) beta-Ketothiolase (2-methylacetoacetyl-coenzyme A thiolase) deficiency: identification of two patients in Israel. *J Inherit Metab Dis* 19(5): 698–699. doi: 10.1007/BF01799849.

Gibson KM, Feigenbaum ASJ (1997) Phenotypically mild presentation in a patient with 2-methylacetoacetyl-coenzyme A (β-keto) thiolase deficiency. *J Inherit Metab Dis* 20(5): 712–713. doi: 10.1023/A:1005390829803.

Grünert SC, Schmitt RN, Schlatter SM, Gemperle-Britschgi C, Balcı MC, Berg V, Çoker M, Das AM, Demirko M, Derks TGJ, Gökçay G, Uçar SK, Konstantopoulou V, Korenke GC, Lotz-Havla AS, Schlune A, Staufner C, Tran C, Visser G, Schwab KO, Fukao T, Sass JO (2017) Clinical Presentation and Outcome in a Series of 32 Patients with 2-Methylacetoacetyl-Coenzyme A Thiolase (MAT) Deficiency. *Mol Genet Metab* 122(1-2): 67–75. doi: 10.1016/j.ymgme.2017.06.012.

Hiyama K, Sakura N, Matsumoto T, Kuhara T (1986) Deficient beta-ketothiolase activity in leukocytes from a patient with 2-methylacetoacetic aciduria. *Clin Chim Acta* 155(2): 189–194. doi: 10.1016/0009-8981(86)90283-4.

Hori T, Yamaguchi S, Shinkaku H, Horikawa R, Shigematsu Y, Takayanagi M, Fukao T (2015) Inborn errors of ketone body utilization. *Pediatr Int* 57(1): 41–48. doi: 10.1111/ped.12585.

Hu CH, Qian QQ, Zhu HM, Sun D, Wu SH, Wu G, Hu JS, Liu ZS (2017) An atypical case of mitochondrial acetoacetyl-CoA thiolase deficiency. *Neurology Asia* 22(2) 165–169.

Köse MD, Canda E, Kağnıcı M, İşgüder R, Ünalp A, Uçar SK, Bahr L, Britschgi C, Sass JO, Çoker M (2016) Two Siblings with Beta-Ketothiolase Deficiency: One Genetic Defect Two Different Pictures. *J Pediatr Res* 3(2): 113–116. doi: 10.4274/jpr.25338.

Law CY, Lam CW, Ching CK, Yau KC, Ho TW, Lai CK, Mak CM (2015) NMR-based urinalysis for beta-ketothiolase deficiency. *Clin Chim Acta* 438: 222–225. doi: 10.1016/j.cca.2014.08.041.

Merinero B, Pérez-Cerdá C, García, MJ, Carrasco S, Lama R, Ugarte M, Middleton B (1987) β-Ketothiolase deficiency: Two siblings with different clinical conditions. *J Inherit Metab Dis* 10 (Suppl 2): 276–278. doi: 10.1007/BF01811425.

Middleton B, Bartlett K, Romanos A, Gomez Vazquez J, Conde C, Cannon RA, Lipson M, Sweetman L, Nyhan WL (1986) 3-Ketothiolase deficiency. *Eur J Pediatr* 144(6): 586–589. doi: 10.1007/BF00496042.

Monastiri K, Amri F, Limam K, Guediche MN (1999) β-Ketothiolase (2-methylacetoacetyl-CoA thiolase) deficiency: a frequent disease in Tunisia?. *J Inher Metab Dis* 22(8): 932–933. doi: 10.1023/A:1005695524913.

Mrázová L, Fukao T, Hálovd K, Gregová E, Kohút V, Pribyl D, Chrastina P, Kondo N, Pospisilová E (2005) Two novel mutations in mitochondrial acetoacetyl-CoA thiolase deficiency. *J Inherit Metab Dis* 28(2): 235–236. doi: 10.1007/s10545-005-7497-6.

Nagasawa H, Yamaguchi S, Orii T, Schutgens RB, Sweetman L, Hashimoto T (1989) Heterogeneity of defects in mitochondrial acetoacetyl-CoA thiolase biosynthesis in fibroblasts from four patients with 3-ketothiolase deficiency. *Pediatr Res* 26(2): 145–149. doi: 10.1203/00006450-198908000-00016.

Nakamura K, Fukao T, Perez-Cerda C, Luque C, Song XQ, Naiki Y, Kohno Y, Ugarte M, Kondo N (2001) A novel single-base substitution (380C>T) that activates a 5-base downstream cryptic splice-acceptor site within exon 5 in almost all transcripts in the human mitochondrial acetoacetyl-CoA thiolase gene. *Mol Genet Metab* 72(2):115–121. doi: 10.1006/mgme.2000.3125.

Nguyen KN, Abdelkreem E, Colombo R, Hasegawa Y, Can NTB, Bui TP, Hai Thanh Le HT, Tran MTC, Nguyen HT, Trinh HT, Aoyama Y, Sasai H, Yamaguchi S, Fukao T, Vu DC (2017) Characterization and outcome of 41 patients with beta-ketothiolase deficiency: 10 years' experience of a medical center in northern Vietnam. *J Inherit Metab Dis* 40(3): 395–401. doi: 10.1007/s10545-017-0026-6.

Otsuka H, Sasai H, Nakama M, Aoyama Y, Abdelkreem E, Ohnishi H, Konstantopoulou V, Sass JO, Fukao T (2016) Exon 10 skipping in ACAT1 caused by a novel c.949G>A mutation located at an exonic splice enhancer site. *Mol Med Rep* 14(5): 4906–4910. doi: 10.3892/mmr.2016.5819.

Ozand PT, Rashed M, Gascon GG, al Odaib A, Shums A, Nester M, Brismar J (1994) 3-Ketothiolase deficiency: a review and four new patients with neurologic symptoms. *Brain Dev* 16(Suppl): 38–45. doi: 10.1016/0387-7604(94)90095-7.

Paquay S, Bourillon A, Pichard S, Benoist JF, de Lonlay P, Dobbelaere D, Fouilhoux A, Guffon N, Rouvet I, Labarthe F, Mention K, Touati G, Valayannopoulos V, de Baulny HO, Acquaviva C, Vianey-Saban C, Schiff M (2017) Mitochondrial acetoacetyl-CoA

thiolase deficiency: basal ganglia impairment may occur independently of ketoacidosis. *J Inherit Metab Dis* 40(3): 415–422. doi: 10.1007/s10545-017-0021-y.

Renom G, Fontaine M, Rolland MO, Duprey J, Degand PM, Dobbelaere D (2000) A new case of 2-methylacetoacetyl-CoA thiolase deficiency?. *J Inherit Metab Dis* 23(7): 751–753. doi: 10.1023/A:10056556.

Riudor E, Ribes A, Perez-Cerda C, Arranz JA, Mora J, Yeste D, Castello F, Christensen B, Sovik O (1995) Metabolic coma with ketoacidosis and hyperglycaemia in 2-methylacetoacetyl-CoA thiolase deficiency. *J Inher Metab Dis* 18(6): 748–749. doi: 10.1007/BF02436766.

Sakurai S, Fukao T, Haapalainen AM, Zhang G, Yamada K, Lilliu F, Yano S, Robinson P, Gibson MK, Wanders RJ, Mitchell GA, Wierenga RK, Kondo N (2007) Kinetic and expression analyses of seven novel mutations in mitochondrial acetoacetyl-CoA thiolase (T2): identification of a Km mutant and an analysis of the mutational sites in the structure. *Mol Genet Metab* 90(4): 370–378. doi: 10.1016/j.ymgme.2006.12.002.

Sarafoglou K, Matern D, Redlinger-Grosse K, Bentler K, Gaviglio A, Harding CO, Rinaldo P (2011) Siblings with mitochondrial acetoacetyl-CoA thiolase deficiency not identified by newborn screening. *Pediatrics* 128(1): e246–e250. doi: 10.1542/peds.2010-3918.

Sasai H, Aoyama Y, Otsuka H, Abdelkreem E, Nakama M, Hori T, Ohnishi H, Turner L, Fukao T (2017) Single nucleotide substitution T to A in the poly-pyrimidine stretch at splice acceptor site of intron 9 causes exon 10 skipping in the *ACAT1* gene. *Mol Genet Genomic Med* 5(2): 177–184. doi: 10.1002/mgg3.275.

Schutgens RB, Middleton B, vd Blij JF, Oorthuys JW, Veder HA, Vulsma T, Tegelaers WH (1982) Beta-ketothiolase deficiency in a family confirmed by in vitro enzymatic assays in fibroblasts. *Eur J Pediatr* 139(1): 39–42. doi: 10.1007/BF00442077

Sewell AC, Herwig J, Wiegratz I, Lehnert W, Niederhoff H, Song XQ, Kondo N, Fukao T (1998) Mitochondrial acetoacetyl-CoA thiolase (β-ketothiolase) deficiency and pregnancy. *J Inher Metab Dis* 21(4): 221–442. doi: 10.1023/A:1005335515166.

Su L, Li X, Lin R, Sheng H, Feng Z, Liu L (2017) Clinical and molecular analysis of 6 Chinese patients with isoleucine metabolism defects: identification of 3 novel mutations in the HSD17B10 and ACAT1 gene. *Metab Brain Dis* 32(6): 2063–2071. doi: 10.1007/s11011-017-0097-y.

Sundaram S, Nair M, Namboodhiri S, Menon RN (2018) Mitochondrial acetoacetyl-CoA thiolase enzyme deficiency in a 9-month old boy: Atypical urinary metabolic profile with a novel homozygous mutation in ACAT1 gene. *Neurol India* 66(6):1802-1804. doi: 10.4103/0028-3886.246264.

Thümmler S, Dupont D, Acquaviva C, Fukao T, De Ricaud D (2010) Different Clinical Presentation in Siblings with Mitochondrial Acetoacetyl-CoA Thiolase Deficiency and Identification of Two Novel Mutations. *Tohoku J Exp Med* 220(1): 27–31. doi: 10.1620/tjem.220.27.

Vakili R, Hashemian S (2018) A Novel Mutation of Beta-ketothiolase Deficiency: The First Report from Iran and Review of Literature. *Iran J Child Neurol* 12(3):113-121. doi: 10.22037/ijcn.v12i3.16645.

Wajner M, Sanseverino MT, Giugliani R, Sweetman L, Yamaguchi S, Fukao T, Shih VE (1992) Biochemical investigation of a Brazilian patient with a defect in mitochondrial acetoacetylcoenzyme-A thiolase. *Clin Genet* 41(4): 202–205. doi: 10.1111/j.1399-0004.1992.tb03663.x.

Wakazono A, Fukao T, Yamaguchi S, Hori T, Orii T, Lambert M, Mitchell GA, Lee GW, Hashimoto T (1995) Molecular, biochemical, and clinical characterization of mitochondrial acetoacetyl-coenzyme A thiolase deficiency in two further patients. *Hum Mutat* 5(1): 34–42. doi: 10.1002/humu.1380050105.

Wen P, Chen Z, Wang G, Su Z, Zhang X, Tang G, Cui D, Liu X, Li C (2016) Analysis of clinical phenotype and ACAT1 gene mutation in a family affected with beta-ketothiolase deficiency. *Chinese Journal of Medical Genetics* 33(3): 286–291. doi: 10.3760/cma.j.issn.1003-9406.2016.03.002.

Wojcik MH, Wierenga JK, Rodan, LH, Sahai I, Ferdinandusse S, Genetti CA, Towne MC, Peake RWA, James PM, Beggs AH, Brownstein CA, Berry GT, Agrawal PB (2018) Beta-Ketothiolase Deficiency Presenting with Metabolic Stroke After a Normal Newborn Screen in Two Individuals. *JIMD Rep* 39:45–54. doi: 10.1007/8904_2017_45.

Yalçinkaya C, Apaydin H, Ozekmekçi S, Gibson KM (2001) Delayed-onset dystonia associated with 3-oxothiolase deficiency. *Mov Disord* 16(2): 372–375. doi: 10.1002/mds.1060.

Yamaguchi S, Orii T, Sakura N, Miyazawa S, Hashimoto T (1988) Defect in biosynthesis of mitochondrial acetoacetyl-coenzyme A thiolase in cultured fibroblasts from a boy with 3-ketothiolase deficiency. *J Clin Invest* 81(3): 813–817. doi: 10.1172/JCI113388.

Yamaguchi S, Fukao T, Kano M, Wakazono A, Orii T, Sakura N, Hashimoto T (1992) Further analysis of mutant thiolase protein in fibroblasts from a Japanese boy with 3-ketothiolase deficiency. *Tohoku J Exp Med* 167(2): 143–153. doi: 10.1620/tjem.167.143.

Yamaguchi S, Sakai A, Fukao T, Wakazono A, Kuwahara T, Orii T, Hashimoto T (1993) Biochemical and immunochemical study of seven families with 3-ketothiolase deficiency: diagnosis of heterozygotes using immunochemical determination of the ratio of

mitochondrial acetoacetyl-CoA thiolase and 3-ketoacyl-CoA thiolase proteins. *Pediatr Res* 33(5): 429–432. doi: 10.1203/00006450-199305000-00001.

Zhang GX, Fukao T, Rolland MO, Zabot MT, Renom G, Touma E, Kondo M, Matsuo N, Kondo N (2004) Mitochondrial acetoacetyl-CoA thiolase (T2) deficiency: T2-deficient patients with "mild" mutation(s) were previously misinterpreted as normal by the coupled assay with tiglyl-CoA. *Pediatr Res* 56(1): 60–64. doi: 10.1203/01.PDR.0000129657.48122.52.

Zhang GX, Fukao T, Sakurai S, Yamada K, Gibson KM, Kondo N (2006) Identification of Alu-mediated, large deletion-spanning exons 2–4 in a patient with mitochondrial acetoacetyl-CoA thiolase deficiency. *Mol Genet Metab* 89(3): 222–226. doi: 10.1016/j.ymgme.2006.06.010.

Supp. Material

The information on the experimental details of the expression and the catalytic assays of *ACAT1* variants associated with mitochondrial acetoacetyl-CoA thiolase (T2) deficiency

Our standard transient expression analysis of T2 cDNA at Gifu University utilizes a pCAGGS eukaryote expression vector, as previously described (Fukao et al., 1998; Niwa et al., 1991; Zhang et al., 2004). In brief, we use a Kod-Plus-Mutagenesis Kit® (Tyobo Co., Osaka, Japan) to design a full-length variant T2 cDNA. Wild-type and variant constructs are transfected via Lipofectamine® 2000 (Invitrogen, San Diego, CA, USA) into 5×10^5 SV40-transformed T2-deficient fibroblasts. Cells are incubated at 37°C (and also at 30°C and 40°C for some variants) for 72 hours, then they are harvested and stored at −80°C until needed. After that, cells are freeze–thawed and sonicated in 50 mM sodium phosphate (pH 8.0) and 0.1% Triton X-100. This is followed by centrifugation at 10,000 × *g* for 10 minutes. Supernatants from cell extracts are used for the expression analysis and enzyme assay. For measuring the expression efficiency an immunoblot analysis is performed as previously described (Fukao et al., 1998; Fukao et al., 1997). Briefly, we use a mixture of an anti-T2 and anti-succinyl-CoA:3-oxoacid CoA transferase (SCOT) polyclonal antibodies as the first antibody. Serial dilution samples extracted from the wild-type are electrophoresed together with mock and variant samples; therefore, we can estimate the amount of any detected variant T2 protein compared to that of wild-type. The expression level of a variant is lower than wild-type in case the variant causes lower folding and/or stability properties.

For the enzyme assay we spectrophotometrically monitor the decrease of the absorbance of the Mg^{2+} complex of acetoacetyl-CoA at 303 nm at a standard temperature of 30°C, which is caused by acetoacetyl-CoA thiolysis to acetyl-CoA. We measure acetoacetyl-CoA thiolase activity in the absence and in the presence of potassium ions (50 mM KCl). Potassium ions specifically stimulate T2; therefore, the difference between these two measurements represents the T2 activity (Fukao et al., 1998; Zhang et al., 2004). We calculate the average and standard errors of three independent experiments.

References

Fukao T, Xiang-Qian S, Yamaguchi S, Kondo N, Orii T, Matthieu JM, Bachmann C, Hashimoto T (1997) Identification of three novel frameshift mutations (83delAT, 754insCT, and 435 + 1G to A) of mitochondrial acetoacetyl-coenzyme

A thiolase gene in two Swiss patients with CRM-negative beta-ketothiolase deficiency. *Hum Mutat* 9(3): 277–279. doi: 10.1002/(SICI)1098-1004(1997)9:33.0.CO;2-#.

Fukao T, Nakamura H, Song XQ, Nakamura K, Orii KE, Kohno Y, Kano M, Yamaguchi S, Hashimoto T, Orii T, Kondo N (1998) Characterization of N93S, I312T, and A333P missense mutations in two Japanese families with mitochondrial acetoacetyl-CoA thiolase deficiency. *Hum Mutat* 12(4): 245–254. doi: 10.1002/(SICI)1098-1004(1998)12:4<245::AID-HUMU5>3.0.CO;2-E.

Niwa H, Yamamura K, Miyazaki J (1991) Efficient selection for high-expression transfectants with a novel eukaryotic vector. *Gene* 108(2): 193–199. doi: 10.1016/0378-1119(91)90434-D.

Zhang GX, Fukao T, Rolland MO, Zabot MT, Renom G, Touma E, Kondo M, Matsuo N, Kondo N (2004) Mitochondrial acetoacetyl-CoA thiolase (T2) deficiency: T2-deficient patients with "mild" mutation(s) were previously misinterpreted as normal by the coupled assay with tiglyl-CoA. *Pediatr Res* 56(1): 60–64. doi: 10.1203/01.PDR.0000129657.48122.52.
